# Supplementary material for: Self-reported sick leave following a brief preventive intervention on work-related stress: a randomised controlled trial in primary health care
Source: BMJ Open. 2021 Mar 22;11(3):e041157. doi: 10.1136/bmjopen-2020-041157 (PMC7986880; doi:10.1136/bmjopen-2020-041157)
Supplement: Author's manuscript [file bmjopen-2020-041157.draft_revisions.pdf]

BMJ Open is committed to open peer review. As part of this commitment we make the peer review history of every article we publish publicly available.

When an article is published we post the peer reviewers' comments and the authors' responses online. We also post the versions of the paper that were used during peer review. These are the versions that the peer review comments apply to.

The versions of the paper that follow are the versions that were submitted during the peer review process. They are not the versions of record or the final published versions. They should not be cited or distributed as the published version of this manuscript.

BMJ Open is an open access journal and the full, final, typeset and author-corrected version of record of the manuscript is available on our site with no access controls, subscription charges or pay-per-view fees (http://bmjopen.bmj.com).

If you have any questions on BMJ Open's open peer review process please email
info.bmjopen@bmj.com

BMJ Open

Self-reported sick leave following a brief preventive intervention on work-related stress: a randomised controlled trial in primary health care

Journal:	*BMJ Open*
Manuscript ID	bmjopen-2020-041157
Article Type:	Original research
Date Submitted by the Author:	02-Jun-2020
Complete List of Authors:	Hultén, Anna-Maria; University of Gothenburg Sahlgrenska Academy,<br>Bjerkeli, Pernilla; University of Skovde<br>Holmgren, Kristina; University of Gothenburg Sahlgrenska Academy
Keywords:	PRIMARY CARE, OCCUPATIONAL & INDUSTRIAL MEDICINE, PUBLIC HEALTH, MEDICAL EDUCATION & TRAINING

SCHOLARONE™
Manuscripts

I, the Submitting Author has the right to grant and does grant on behalf of all authors of the Work (as defined in the below author licence), an exclusive licence and/or a non-exclusive licence for contributions from authors who are: i) UK Crown employees; ii) where BMJ has agreed a CC-BY licence shall apply, and/or iii) in accordance with the terms applicable for US Federal Government officers or employees acting as part of their official duties; on a worldwide, perpetual, irrevocable, royalty-free basis to BMJ Publishing Group Ltd ("BMJ") its licensees and where the relevant Journal is co-owned by BMJ to the co-owners of the Journal, to publish the Work in this journal and any other BMJ products and to exploit all rights, as set out in our licence.

The Submitting Author accepts and understands that any supply made under these terms is made by BMJ to the Submitting Author unless you are acting as an employee on behalf of your employer or a postgraduate student of an affiliated institution which is paying any applicable article publishing charge ("APC") for Open Access articles. Where the Submitting Author wishes to make the Work available on an Open Access basis (and intends to pay the relevant APC), the terms of reuse of such Open Access shall be governed by a Creative Commons licence – details of these licences and which Creative Commons licence will apply to this Work are set out in our licence referred to above.

Other than as permitted in any relevant BMJ Author's Self Archiving Policies, I confirm this Work has not been accepted for publication elsewhere, is not being considered for publication elsewhere and does not duplicate material already published. I confirm all authors consent to publication of this Work and authorise the granting of this licence.

For peer review only

Self-reported sick leave following a brief preventive intervention on work-related stress: a randomised controlled trial in primary health care

Anna-Maria Hultén[a]*, e-mail: anna-maria.hulten@gu.se

Pernilla Bjerkeli[a,b], e-mail: pernilla.bjerkeli@his.se

Kristina Holmgren[a], e-mail: kristina.holmgen@neuro.gu.se

[a]Unit of Occupational Therapy, Department of Health and Rehabilitation, Institute of Neuroscience and Physiology, Sahlgrenska Academy, University of Gothenburg, Box 455, 405 30 Gothenburg, Sweden

[b]Department for Public Health Research, University of Skövde, Box 408, Skövde, 541 28, Sweden

*Corresponding author

For peer review only

ABSTRACT

Objectives: To evaluate the effectiveness of a brief intervention about early identification of work-related stress combined with feedback at consultation with a general practitioner (GP) on the number of self-reported sick leave days.

Design: Randomised controlled trial. Prospective analyses of self-reported sick leave data collected between November 2015 and January 2017.

Setting: Seven primary health care centres in western Sweden.

Participants: The study included 271 employed, non-sick-listed patients aged 18–64 years seeking care for mental and/or physical health complaints. Of these, 132 patients were allocated to intervention and 139 patients to control.

Interventions: The intervention group received a brief intervention about work-related stress, including training for GPs, screening of patients' work-related stress, feedback to patients on screening results and discussion of measures at GP consultation. The control group received treatment as usual.

Outcome measures: The number of self-reported gross sick leave days and the number of self-reported net sick leave days.

Results: Separate analyses were performed for 6 and 12 months' follow-up and for five different subsamples. The results indicate that there was no significant difference between the intervention group and the control group.

Conclusions: The brief intervention showed no effect on the numbers of self-reported sick leave days for patients seeking care at the primary health care centres. Other actions and new types of interventions need to be explored to address patients' perceiving of ill health due to work-related stress.

Trial registration number: NCT02480855, ClinicalTrials.gov.

For peer review only - http://bmjopen.bmj.com/site/about/guidelines.xhtml

STRENGTHS AND LIMITATIONS OF THIS STUDY

- Few previous RCTs have focused on patients' sick leave in a primary health care context.
- Using self-reported sick leave data made it possible to include the first two weeks of sick leave, which are not included in register data.
- Due to the inherent complexity in clinical trials in primary health care practice, the statistical power of the study might have been low.
- Sick leave data are not normally distributed and non-parametric tests therefore had to be used for the analysis.
- The outcome measure (sick leave days) is complex to interpret, as it is used both as an indicator for ill health and as a tool for treatment of ill health.

For peer review only

INTRODUCTION

Work-related stress has been in focus for decades, as it is common and affects the individual and the society in multiple ways. Depression, anxiety and musculoskeletal disorders are all possible consequences of work-related stress.[1 2] Psychosocial work conditions and work-related stress also constitute risk factors for sick leave.[1] As a consequence, almost 50% of the 3 billion EUR paid for sickness benefits in Sweden in 2018 were due to mental disorders,[3] whereof reaction to severe stress and adjustment disorders constituted half,[4] not to mention the loss of working hours and the costs for treatment and rehabilitation.

Sick leave is a common outcome measure in research. However, the relationship between spells, morbidity and health is complex, since sick leave is influenced strongly by factors other than personal health.[5-7] Hence, controversy exists about how to conceptualise sick leave in research.[6] As individual, social and economic forces jointly determine absence behaviour, aspects other than work-related stress must be considered, such as attendance motivation, absence culture and sickness benefit reform.[6-8] Even so, sick leave can be a useful measure not only of health status and functioning[9] but also of future sick leave and use of disability pension.[10 11] In addition, using self-reported sick leave data makes it possible to consider the first two weeks of absence, which are not included in the Swedish social insurance agency's register data.

Research has shown that there is a strong correlation between sick leave and work-related stress[12 13] and that early identification of persons perceiving ill health is important for preventing sick leave.[11 14] In addition, screening for interacting individual and work factors could make it possible to focus on the patient's specific problems and aid in finding suitable treatments.[15] In Sweden, primary health care is responsible for basic medical treatment, nursing, preventive work and rehabilitation that do not require the medical and technical resources of a hospital or other specialist skills.[16] Primary health care is also considered best suited for preventive work.[16] Since general practitioners (GPs) are often the first health care contact for persons having physical or mental health complaints

For peer review only - http://bmjopen.bmj.com/site/about/guidelines.xhtml

and often handle cases concerning stress and work ability,[17 18] they could be a possible starting point for preventive actions concerning ill health due to work-related stress.

Commonly, GPs working at a primary health care centre in Sweden have access to several other healthcare professionals, such as nurses, occupational therapists, physiotherapists and social workers, sometimes organised in psychosocial teams.[19] However, the proportion of GPs is lower than for most other comparable high-income countries, as are investments in other primary care resources.[20] In addition, earlier studies have shown that GPs might not have the prerequisites needed for early identification and treatment of patients perceiving ill health due to work-related stress in order to decrease sick leave.[21-23] Therefore, a brief preventive intervention was designed using the Work Stress Questionnaire (WSQ)[24 25] as a screening tool in combination with feedback at patient–GP consultations.[26]

METHOD

This two-armed non-blinded randomised controlled trial (RCT) was conducted at primary health care centres (PHCC) located in both urban and rural areas in the region Västra Götaland in Sweden. The trial has been previously described in detail in a study protocol[26] and in a research article.[27]

Objectives

The objective of the study was to evaluate the effectiveness of the brief intervention about early identification of work-related stress combined with feedback at GP consultation on the number of self-reported sick leave days. The overall hypothesis was that the intervention group would have fewer sick leave days during the year after the brief intervention compared to the control group. The assumptions behind this were that (1) taking part in an initial training session increased the GPs' knowledge on work-related stress, (2) filling in the WSQ raised the patients' awareness about their level of work-related stress through self-reflection, (3) receiving feedback on WSQ results increased the patients' motivation to address their work situation and (4) the combined effect of the training

For peer review only - http://bmjopen.bmj.com/site/about/guidelines.xhtml

For peer review only

session, filling in the WSQ and receiving feedback constituted a basis for in-depth discussions on relevant measures at the GP–patient consultation.

The intervention concerned sick leave due to work-related stress. Hence, it was assumed that the effect of the intervention was higher for patients reporting high work-related stress or high exposure to stressors according to the WSQ. This group was therefore studied explicitly.

Procedure

Seven PHCCs were included in the study, of which four were public and three were privately run. Participating GPs had to be working at least 50% of the time at the PHCC. The recruitment of patients and the performance of the interventions were conducted in parallel for a period of 4–12 weeks at each PHCC from May 2015 until January 2016. Before the intervention period, the research team visited the centre to inform the staff about the study. During the intervention period, a research assistant was stationed at the PHCC to identify and recruit the eligible participants, give information on the study and administer patients' informed consent. In addition, extra personnel resources were needed to perform the training session and to administer the WSQ to the patients.

Intervention

As an initial step, the GPs randomised to intervention received a two-hour training session including information about work-related stress, ill health and sick leave. Instructions were also given on how to use the WSQ and how to give feedback to the participants; in addition, GPs received information on healthcare professionals available for referral. Before the GP–patient consultation, each patient filled in the WSQ and questions on background characteristics. During consultation, the intervention GPs gave feedback to the patients on the WSQ results. In addition, the GP and patient conferred about and initiated preventive measures, if needed.

Control

For peer review only - http://bmjopen.bmj.com/site/about/guidelines.xhtml

The GPs randomised to control were instructed to carry on as usual with their consultations and were not informed as to whether or not the patients were participating in the study. After the consultation, the control patients filled in the WSQ and gave information about background characteristics.

Outcomes

The study had two primary outcome measures: 1. Number of self-reported gross sick leave days and 2. Number of self-reported net sick leave days. The measures were based on the following request at follow-up: Define your sick leave during the latest 3 or 6 months, each occasion separately (number of days with sick leave and extent of sick leave per occasion: 0%, 25%, 50%, 75%, 100% or varying extent). A reported varying extent of sick leave was treated as a 50% sick leave.

Follow-up data were collected at 6 and 12 months after the intervention by telephone or email. At 6 months' follow-up the prior 3 months were reported, while at 12 months' follow-up the prior 6 months were reported. Data for the two follow-ups were treated separately. The number of self-reported gross sick leave days for each follow-up was calculated as the sum of the number of self-reported sick leave days for each sick leave occasion during these months. To calculate the number of self-reported net sick leave days for each follow-up, the self-reported days of sick leave for each occasion was multiplied by the corresponding extent of sick leave and summarised.

The variable of self-reported gross sick leave days was categorised into four levels: 0, 1–7, 8–14 and 15 days and above. These categories were based on the Swedish sickness insurance scheme[28] stating that the employer pays sick pay for up to two weeks, with one qualifying day. Thereafter, sickness benefits are handled by the Swedish Social Insurance Agency. From day 8 of sickness onward, a doctor's certificate is required.

Target group, sample size and power

Patients eligible to participate had to be employed, non-sick-listed, 18–64 years of age and seeking care for mental and/or physical health complaints. The PHCCs were economically compensated for

For peer review only - http://bmjopen.bmj.com/site/about/guidelines.xhtml

each participant recruited. An a priori power analysis was performed to detect at least a 15% difference between the intervention group and the control group concerning the primary outcome, that is, the number of registered sick leave days (14 days or more) during 12 months after inclusion. With a two-sided test, statistical significance of $p<0.05$ and 80% power, at least 135 participants were needed in each group.

Randomisation and blinding

The GPs at the participating PHCCs were randomised to either the intervention group or the control group with a 1:1 allocation. Folded slips of paper with their written names were mixed in a non-transparent bowl and subsequently drawn, one at a time, to the two groups alternately by colleagues not involved in the RCT. The patients consulting the GPs were therefore automatically allocated to either group. Due to the setup of the trial, none of the parties involved were blinded after assignment to interventions. All patients received the study information provided by the research assistant, the intervention GPs received information and training before the study started and the control GPs received information about the study but no training.

Statistical analyses

Outcome data were missing for some patients, due to non-response at follow-up. Therefore, a comparison was made to test whether there were differences in characteristics between patients taking part at 6 and 12 months' follow-up, respectively, and the participants at baseline. Differences in gender proportion, age and health status were tested using chi-square test. As no statistically significant differences were observed, the patients taking part at the follow-up were included in the main analysis. A descriptive analysis was then performed for the categorised length of self-reported gross sick leave, to get an overall understanding of the distribution of sick leave.

For the main analysis, a comparison between the intervention and control groups was made for the gross and net numbers of sick leave days at each follow-up. As the distribution strongly deviated from a normal distribution, the Mann-Whitney U test was used. Additional analyses were

For peer review only

For peer review only - http://bmjopen.bmj.com/site/about/guidelines.xhtml

conducted on five subsamples with patients highly exposed to stressors. The subsamples were identified based on the results from the WSQ,[24] which is a self-assessment questionnaire developed for early identification of people with work-related stress at risk for sick leave. The WSQ is divided into four dimensions with a total of 21 questions concerning influence at work, work organisation and conflicts, and individual demands and commitment as well as interference between work and leisure time. The subsamples were defined as follows:

1. *Low influence at work* included patients' seldom or never perceiving influence at work.
2. *High stress due to indistinct organisation and conflicts* included patients perceiving their work organisation and occurring conflicts as stressful or very stressful.
3. *High stress due to individual demands and commitment* included patients perceiving their own work demands and commitment as stressful or very stressful.
4. *High work to leisure time interference* included patients always or rather often perceiving interference between work and leisure.
5. *Effect from one subsample or more* included participants belonging to at least one of the above-described subsamples 1–4.

All answers were given on a four-point ordinal scale. A missing value in a dimension was replaced by the participant's median for that dimension, but only if there were answers to at least 50% of the questions in the dimension. The median values for each dimension were then categorised into high and low. All statistical analyses were performed in IBM SPSS Statistics 25.

Patient and public involvement statement

There was no patient or public involvement in the planning or conduct of this trial.

RESULTS

Participant flow

For peer review only - http://bmjopen.bmj.com/site/about/guidelines.xhtml

For peer review only

The 66 eligible GPs at the seven PHCCs were randomised to the intervention group or the control group, Figure 1. Since three GPs declined to participate or did not have patients fulfilling the criteria, there were 29 intervention GPs and 34 control GPs included. Following recruitment, 139 patients were allocated to the intervention group and 162 patients to the control group. Of these, 7 patients in the intervention group and 23 in the control group were excluded due to patients declining to participate or due to logistic reasons. Altogether, 271 patients received treatment (intervention n=132 and control n=139). Independent of group allocation, 51/271 (19%) participating patients were lost to the 6-month follow-up and 30/271 (11%) to the 12-month follow-up. Of these, 13/271 (5%) did not participate in either of the follow-ups. At 6 months' follow-up data from 220 patients were included in the main analysis, while at 12 months' follow-up data from 241 patients were included. A flowchart for the enrolment, allocation and follow-ups is presented in Figure 1.

Insert Figure 1

Figure 1 *Flowchart of enrolment, allocation and follow up.*

Baseline data

As shown in Table 1, the intervention group and the control group had similar distribution of background characteristics at baseline (n=271). However, the participants in the intervention group were slightly older and sought care for musculoskeletal ill health to a higher extent.

Results from the WSQ showed that 40% of the patients assessed their influence at work as low, independent of group. Approximately 20% of the patients reported high stress due to indistinct organisation and conflicts, while slightly fewer than 50% reported high stress due to high individual demands and work commitment. The fourth dimension, interference of work with leisure time, was high for 40% of the patients. Finally, 70% of the patients had stressors or stress from at least one of the four dimensions (effect from one subsample or more).

For peer review only - http://bmjopen.bmj.com/site/about/guidelines.xhtml

Table 1 Baseline characteristics of the 271 patients included in the randomised controlled trial and allocated to the intervention group or the control group.

Variable		Total (n=271)		Intervention (n=132)		Control (n=139)	
		n	(%)	n	(%)	n	(%)
Sex	Male	86	32	44	33	42	30
	Female	185	68	88	67	97	70
Age (years)	18–30	47	17	21	16	26	19
	31–50	134	50	58	44	76	54
	51–64[1]	90	33	53	40	37	27
Occupational class	Skilled/unskilled manual	107	40	49	37	58	42
	Medium/low non-manual	116	43	60	46	56	40
	High-level non manual	47	17	23	17	24	17
	Missing	1	0			1	1
Overall health, self-rated[2]	Excellent/very good	77	28	34	26	43	30
	Good	108	40	53	40	55	40
	Satisfactory/unsatisfactory	73	27	39	30	34	25
	Missing	13	5	6	4	7	5
Reason for consultation[3]	Mental or behavioural	144	53	75	57	69	50
	Musculoskeletal[1]	106	39	62	47	44	32
	Gastrointestinal	54	20	26	20	28	20
	Cardiovascular	32	12	16	12	16	13
	Other	56	21	29	22	27	19
WSQ results[4]	Low influence at work	108	40	54	41	54	39
	High stress organisation and conflicts	54	20	28	21	26	19
	High stress demands and work commit.	124	46	63	48	61	44
	High work to leisure time interference	109	40	54	41	55	40
	Effect from one subsample or more	188	69	91	69	97	70

[1]Statistically significant differences (tested with a 95% confidence interval for difference in proportion between case and control)
[2]Short Form Health Survey, SF-36[29]
[3]More than one reason for consultation was possible
[4]Work Stress Questionnaire results from the four dimensions dichotomized into high and low levels as well as from the summary variable including effect from at least one dimension

Analysis of participants responding at follow-up

The basic characteristics of the participants in the intervention and the control groups responding at follow-up are shown in Table 2. No statistically significant differences were found between baseline and responders at 6 and 12 months' follow-up concerning sex, age or self-rated health.

Table 2 Characteristics of participants responding in the intervention group and the control group at 6 and 12 months' follow-up compared to baseline.

Variable, 6 months (n=220)		Intervention			Control		
		Baseline	Respond part[1]	p-value[2]	Baseline	Respond part[1]	p-value[2]
Numbers		132	105		139	115	
Sex	Male	44	33	0.756	42	35	0.970
	Female	88	72		97	80	
Age (years)	18–30	21	17	0.950	26	23	0.908
	31–50	58	44		76	64	
	51–64	53	44		37	28	
Overall	Excellent/very good	34	26		43	36	

For peer review only - http://bmjopen.bmj.com/site/about/guidelines.xhtml

health, self-rated[3]	Good	53	39	0.807	55	45	0.988
	Satisfactory/unsatisfactory	39	35		34	27	
	Missing	6	5		7	7	
**Variable, 12 months (n=241)**		**Intervention**			**Control**		
		**Baseline**	**Respond-ers[4]**	**p-value[2]**	**Baseline**	**Respond-ers[1]**	**p-value[2]**
Numbers		132	119		139	122	
Sex	Male	44	39	0.925	42	40	0.655
	Female	88	80		97	82	
Age (years)	18–30	21	20	0.951	26	24	0.869
	31–50	58	50		76	69	
	51–64	53	49		37	29	
Overall health, self-rated[3]	Excellent/very good	34	32	0.968	43	38	0.968
	Good	53	46		55	49	
	Satisfactory/unsatisfactory	39	35		34	28	
	Missing	6	6		7	7	

[1]6 months' follow-up
[2]Testing the distribution between baseline and responders at 6 months' follow-up concerning sex, age and health with Pearson's chi-2 test
[3]Short Form Health Survey, SF-36[29]
[4]12 months' follow-up

Descriptive statistics of sick leave

As shown in Figure 2, 59/105 in the intervention group and 61/115 in the control group reported no sick leave at the 6-month follow-up. At the 12-month follow-up the corresponding numbers were 61/119 and n=57/122, respectively.

Insert Figure 2

Figure 2 *Total days of sick leave per individual at 6 months' follow-up (n=105 in the intervention group and n=115 in the control group) and at 12 months' follow-up (n=119 in the intervention group and n=122 in the control group).*

Main analysis of sick leave

The main analysis included 220 participants at 6 months' follow-up and 241 participants at 12 months' follow-up (Figure 1). The median and quartiles for both gross and net sick leave days are shown in Table 3. There was no statistically significant difference between the intervention group and the control group.

Sick leave in subsamples

For peer review only - http://bmjopen.bmj.com/site/about/guidelines.xhtml

For peer review only

The comparison of numbers of gross sick leave days for the five subsamples is shown in Table 3. There were no statistically significant differences between the intervention group and the control group for any of these groups.

Table 3 Comparison of sick leave days between the intervention group and the control group at 6 and 12 months' follow-up including analysis for five subsamples.

Follow-up	Sick leave	Group	Quartiles 25	50	75	p-value[1]
6 months, (n=220)	Gross days	Intervention	0.0	0.0	6.0	0.449
		Control	0.0	0.0	10.0	
	Net days	Intervention	0.0	0.0	5.9	0.398
		Control	0.0	0.0	9.0	
12 months, (n=241)	Gross days	Intervention	0.0	0.0	7.0	0.505
		Control	0.0	1.0	7.2	
	Net days	Intervention	0.0	0.0	6.2	0.490
		Control	0.0	1.0	6.2	
**Subsamples**	**Sick leave**	**Group**	**Quartiles 25**	**50**	**75**	**p-value[1]**
Low influence	Gross days 6 months, (n= 89)	Intervention	0.0	1.0	10.0	0.810
		Control	0.0	0.5	27.0	
	Gross days 12 months (n= 94)	Intervention	0.0	2.0	7.0	0.916
		Control	0.0	2.0	6.0	
Stress due to organisation and conflicts	Gross days 6 months (n=45)	Intervention	0.0	0.0	7.5	0.931
		Control	0.0	0.0	17.5	
	Gross days 12 months (n=47)	Intervention	0.0	2.5	7.7	0.877
		Control	0.0	2.0	12.0	
Stress due to commitment	Gross days 6 months (n=103)	Intervention	0.0	1.0	14.5	0.793
		Control	0.0	0.0	10.2	
	Gross days 12 months (n=106)	Intervention	0.0	2.0	8.0	0.321
		Control	0.0	0.0	5.0	
Work to leisure time interference	Gross days 6 months (n=89)	Intervention	0.0	0.0	6.5	0.446
		Control	0.0	0.0	30.0	
	Gross days 12 months (n=96)	Intervention	0.0	2.0	10.0	0.296
		Control	0.0	0.0	5.0	
Effect, any dimension	Gross days 6 months (n=154)	Intervention	0.0	0.0	8.0	0.492
		Control	0.0	0.0	19.0	
	Gross days 12 months (n=164)	Intervention	0.0	2.0	8.7	0.310
		Control	0.0	1.0	5.7	

[1]Mann-Whitney U test

DISCUSSION

Principal findings

This study investigated differences in self-reported sick leave between patients receiving a brief intervention to prevent sick leave due to work-related stress and those receiving treatment as usual. The results indicate that there was no significant difference in self-reported sick leave between the

For peer review only - http://bmjopen.bmj.com/site/about/guidelines.xhtml

intervention group and the control group at 6 and 12 months' follow-up. This is in line with earlier findings from the same RCT using sick leave data from a national Swedish register including only spells 15 days and above.[27] Further, there were no significant differences in the subsamples, that is, among patients highly exposed to stressors.

Interpretation of findings

In this study, sick leave is used as an outcome measure, as it is considered a useful integrated measure of physical, psychological and social functioning in studies of working populations.[9] However, the relationship between ill health and sick leave is complex,[7 29] since it includes absence from work that is attributed to sickness by the employee and accepted as such by the employer[5] and other actors. To some extent, sick leave reflects employees' perceptions of their health and their behaviour in response to ill health.[9] Ill health can therefore be treated as a prerequisite of sick leave seen in relation to conditions within and outside of work.[30] Thus, previous intervention studies on sick leave have not demonstrated any effect on sick leave.[31-33] Further, short-term sick leave is considered to be more influenced by social, legal and psychological factors than health compared to long-term sick leave.[8 9] An essential component of the brief intervention was the discussion of relevant preventive measures during consultation. In general, GPs regard sickness certification as a powerful and important tool.[34] In addition, workers use sick leave as a form of self-medication and a preventive measure when perceiving strain at work.[35] Hence, the brief intervention might have contributed to GPs and patients using short-term sick leave as an early treatment and as a preventive measure to a higher extent than otherwise. Since sick leave is used both as an indicator for ill health and as a tool for treatment of ill health, an initial reduction in sick leave might not be a positive outcome of the brief intervention. This complexity might be a reason why the number of sick leave days was not lower for the intervention group than the control group.

The layout of the brief intervention is fundamental for the results retrieved. The first and perhaps foremost aspect of the intervention was to increase the GPs' knowledge and awareness about work-related stress, but the training session received might not have been exhaustive enough

For peer review only - http://bmjopen.bmj.com/site/about/guidelines.xhtml

to raise GPs' attention to patients with work-related problems or lead them to address such a complex health issue.[36 37] Secondly, filling in the WSQ was expected to increase the patients' awareness about their symptoms being stress-related. The use of patient-reported outcome measures has indeed been shown to improve the understanding of symptoms and facilitate communication.[38 39] However, early in the clinical reasoning process patients could be in need of rapport building and exclusion of physical diseases and consequently resist a psychiatric explanation.[40] Thirdly, receiving feedback on WSQ results was hypothesised to increase patients' motivation to address their work situation. However, the link between antecedents of motivation and enactment is complex. It is therefore necessary to take, for instance, past behaviour, intention, perceived behavioural control and outcome expectancy into account[41] to be able to understand this link. Thus, receiving feedback might not be sufficient to increase motivation to act. Fourthly, the first three components combined in the brief intervention were assumed to constitute a basis for fruitful GP–patient discussions and initiating relevant measures. In concordance, collaborations with patients and colleagues are seen as important elements in the referral process.[42] However, according to GPs, other aspects such as reluctance to cooperate with patients and sparse contact with colleagues could affect the referral process[42] and the measures taken. Taken together, factors related to the study setup might have diluted the effect of the intervention, so that no difference in self-reported sick leave days was detected, even for the subsamples highly exposed to stressors.

The last step of the brief intervention, that is, discussing measures, was left for the GPs to organise as they deemed fit, rather than being specified in the study protocol. In general, GPs have a common understanding of their practice arising not only from the field of general practice but also from the mission of the Swedish primary health care system.[19] The overall way of working would therefore be similar. However, the results from a process evaluation of this RCT (Hultén, Dahlin-Ivanoff, Holmgren. Positioning work-related stress: GPs' reasoning about using the WSQ combined with feedback at consultation, in preparation) indicate that the prerequisites for discussing measures might not have been ideal. The brief intervention was not found to assist the GPs in their work, since

For peer review only - http://bmjopen.bmj.com/site/about/guidelines.xhtml

it could alter their already well-functioning work procedure. This confirms previous findings, where the use of instruments to obtain a quantitative score of depression was not perceived as useful by GPs.[43] The process evaluation also showed that the GPs could find it difficult to interpret and act on the results from the WSQ and could even question their responsibility for prevention of patients' ill health due to work-related stress, when resources were sparse. The intervention might therefore not have been efficient enough to add any effect on the days of sick leave at the follow-ups. Further, these aspects might have diminished the differences in measures taken between the intervention group and the control groups.

Strengths and limitations

Few RCTs in primary health care have focused on patients' sick leave.[31-33] In some respects, this study can be considered as pragmatic, since it is designed to test the impact of the brief intervention on sick leave in clinical practice. Inherent in pragmatic trials is a significant heterogeneity concerning patients, treatments and clinical settings, which leads to dilution of the effect of the intervention.[44] Consequently, pragmatic trials must be large. The lack of statistical difference between the intervention group and the control group could therefore be caused by a lack of statistical power due to a small sample size. The trial also included aspects of explanatory trials, that is, trials that aim to evaluate the efficacy of an intervention in a well-defined and controlled setting,[44] as extra personnel administered parts of the intervention. Otherwise, the study would not have been feasible. As a result, the generalisability and application in routine practice settings decreased.

To find the patients at risk of sick leave due to work-related stress, the inclusion criteria had to be wide. The target group of the study might have included patients not perceiving work-related stress, that is, patients not in need of the intervention. Apart from the main analysis, additional analyses were therefore performed for five subsamples with patients highly exposed to stressors. However, the sample size and power thereby decreased.

The choice of outcome measures has to be taken into consideration. There are different methodological aspects and approaches to consider in using sick leave data in research,[45] Spell

For peer review only - http://bmjopen.bmj.com/site/about/guidelines.xhtml

measures, person measures and time-based measures have to be used wisely[45] to capture any differences between the intervention group and the control group. Therefore, both the self-reported gross sick leave days and net sick leave days were used as outcome measures in this study. However, other outcome measures describing sick leave, such as not only number of days from intervention to sick leave but also health-related measures, might have been needed to capture an effect of the intervention.

The use of self-reported sick leave data was considered as a reasonable choice, as it made it possible to account for the first two weeks of sick leave. Thereby, any short periods of sick leave initiated by the workers themselves[35] or by the GPs were included. Even so, self-reported data can be afflicted with recall bias. However, earlier studies indicate that there is good agreement between self-reported data and register information.[46 47] Even though the response rate was high, data were missing. Non-responders had to be accounted for, as this could affect the validity of trial findings.[48] Multiple imputation of missing data was not possible, since the data were not normally distributed. In addition, simple imputation, such as last value carried forward, was found to be inappropriate, as it assumes a strong correlation between a prior and a later value. Since there were no statistically significant differences in characteristics between responders at baseline and at follow-up, using not imputed data for responders at 6 and 12 months' follow-up for the main analysis was considered the best option. In addition, analysing sick leave data can be challenging, as it is not normal distributed.[45] Non-parametric tests, generally with less power, were therefore used in this study. The relatively small sample size and the statistical methods used both contributed to lowering the power. Thus, it is not possible to know whether the intervention had no effect or if it was not possible to detect an effect.

Conclusions and implications

Based on the results from this RCT, the brief intervention showed no effect on the number of self-reported sick leave days. However, the study yielded information about the provision of

For peer review only - http://bmjopen.bmj.com/site/about/guidelines.xhtml

interventions in primary health care. When performing RCTs in primary health care settings, the design is determined by what is regarded as viable. Contextual aspects such as adapted educational efforts on different levels, the patients' needs and GPs' attitudes to the intervention have to be considered thoroughly when developing and implementing interventions on preventing sick leave due to work-related stress. In addition, the results can lead to discussions about how to use sick leave as an outcome measure. Even so, there is a significant need for further research into these issues, given the individual and societal consequences of ill health due to work-related stress and the limited resources to provide treatment in a cost-effective way.

DECLARATIONS

Acknowledgements The authors would like to acknowledge the PHCCs and especially the GPs and patients taking part in this study as well as the co-workers in the research group TIDAS for their support and feedback.

Contributors KH is the principal investigator and in charge of the RCT. KH was involved in designing the RCT and applying for funding. Statistical analysis was performed by AMH in close collaboration with PB. AMH drafted the manuscript, which was edited by KH and PB. All three authors critically reviewed and approved the final version of the manuscript.

Funding This study was funded by the Swedish Research Council for Health, Working Life and Welfare (2014-0936).

Disclaimer The Swedish Research Council for Health, Working Life and Welfare had no role in the design of the study, data collection, analysis or interpretation of data, or in writing the manuscript.

Competing interests The authors declare that they have no competing interests.

Patient consent for publication Not required.

For peer review only

Ethical approval The project received ethical approval, reference number 125–15, from the Regional Ethical Review Board in Gothenburg, Sweden.

Data availability statement For ethical reasons the datasets generated and analysed during the current study are not publicly available, but they are available from the corresponding author on reasonable request.

Word count 4152 (Introduction, Method, Results, Discussion and Conclusions and implications)

License statement Other than as permitted in any relevant BMJ Author's Self Archiving Policies, I confirm this Work has not been accepted for publication elsewhere, is not being considered for publication elsewhere and does not duplicate material already published. I confirm all authors consent to publication of this Work and authorise the granting of this licence

REFERENCES

1. Mather L, Bergström G, Blom V, et al. High job demands, job strain, and iso-strain are risk factors for sick leave due to mental disorders a prospective Swedish twin study with a 5-year follow-up. *J Occup Environ Med* 2015;57(8):858-65.

2. Da Costa BR, Vieira ER. Risk factors for work-related musculoskeletal disorders: A systematic review of recent longitudinal studies. *Am J Ind Med* 2010;53(3):285-323.

3. Socialförsäkringen i siffror 2019 [Social Insurance in Figures 2019]. Stockholm: The Swedish Social Insurance Agency, 2019.

4. Sjukskrivning för reaktioner på svår stress ökar mest [Sick leave for reactions to severe stress increases most]. Stockholm: The Swedish Social Insurance Agency, 2016.

5. Whitaker SC. The management of sick leave. *Occup Environ Med* 2001;58(6):420-24.

6. Allebeck P, Mastekaasa A. Chapter 3. Causes of sick leave: Research approaches and explanatory models. *Scand J Public Health Suppl* 2004;32(63):36-43.

For peer review only - http://bmjopen.bmj.com/site/about/guidelines.xhtml

For peer review only

7. Kangas M, Muotka J, Huhtala M, et al. Is the Ethical Culture of the Organization Associated with Sick leave? A Multilevel Analysis in a Public Sector Organization. *J Bus Ethics* 2017;140(1):131-45.

8. Aaviksoo E, Kiivet RA. Influence of the sickness benefit reform on sick leave. *Health policy* 2016;120(9):1070-78.

9. Marmot M, Feeney A, Shipley M, et al. Sick leave as a measure of health status and functioning: From the UK Whitehall II study. *J Epidemiol Community Health* 1995;49(2):124-30.

10. Kivimäki M, Forma P, Wikström J, et al. Sick leave as a risk marker of future disability pension: The 10-town study. *J Epidemiol Community Health* 2004;58(8):710-11.

11. Hultin H, Lindholm C, Malfert M, et al. Short-term sick leave and future risk of sick leave and unemployment - The impact of health status. *BMC Public Health* 2012;12(1).

12. Bakker AB, Demerouti E, de Boer E, et al. Job demand and job resources as predictors of absence duration and frequency. *J Vocat Behav* 2003;62(2):341-56.

13. Holmgren K, Hensing G, Dellve L. The association between poor organizational climate and high work commitments, and sick leave in a general population of women and men. *J Occup Environ Med* 2010;52(12):1179-85.

14. Kant I, Jansen NWH, Van Amelsvoort LGPM, et al. Structured early consultation with the occupational physician reduces sick leave among office workers at high risk for long-term sick leave: A randomized controlled trial. *J Occup Rehabil* 2008;18(1):79-86.

15. Boersma K, Linton SJ. Screening to identify patients at risk: Profiles of psychological risk factors for early intervention. *Clin J Pain* 2005;21(1):38-43.

16. God och nära vård – En primärvårdsreform [Good quality, local health care – A primary care reform]. Swedish Government Official Reports, SOU 2018:39. Stockholm: Government Offices of Sweden, Ministry of Health and Social Affairs, 2018. https://data.riksdagen.se/fil/FD4442D3-C4D5-4799-869C-A00930A003F5

For peer review only

For peer review only - http://bmjopen.bmj.com/site/about/guidelines.xhtml

17. Lykkegaard J, Rosendal M, Brask K, et al. Prevalence of persons contacting general practice for psychological stress in Denmark. *Scand J Prim Health Care* 2018;36(3):272-80.

18. Lofgren A, Hagberg J, Arrelov B, et al. Frequency and nature of problems associated with sickness certification tasks: a cross-sectional questionnaire study of 5455 physicians. *Scand J Prim Health Care* 2007;25(3):178-85.

19. Primärvårdens uppdrag : en kartläggning av hur landstingens uppdrag till primärvården är formulerade. Stockholm: The National Board of Health and Welfare, 2016.

20. Anell A. The public-private pendulum-patient choice and equity in Sweden. *New Engl J Med* 2015;372(1):1-4.

21. Sturesson M, Edlund C, Fjellman-Wiklund A, et al. Work ability as obscure, complex and unique: Views of Swedish occupational therapists and physicians. *Work* 2013;45(1):117-28.

22. Nilsing E, Söderberg E, Berterö C, et al. Primary healthcare professionals' experiences of the sick leave process: A focus group study in Sweden. *J Occup Rehabil* 2013;23(3):450-61.

23. Carlsson L, Lännerström L, Wallman T, et al. General practitioners' perceptions of working with the certification of sick leaves following changes in the Swedish social security system: A qualitative focus-group study. *BMC Fam Pract* 2015;16(1)

24. Holmgren K, Hensing G, Dahlin-Ivanoff S. Development of a questionnaire assessing work-related stress in women - Identifying individuals who risk being put on sick leave. *Disabil Rehabil* 2009;31(4):284-92.

25. Frantz A, Holmgren K. The Work Stress Questionnaire (WSQ) - Reliability and face validity among male workers. *BMC Public Health* 2019;19(1).

26. Holmgren K, Sandheimer C, Mardby AC, et al. Early identification in primary health care of people at risk for sick leave due to work-related stress - study protocol of a randomized controlled trial (RCT). *BMC Public Health* 2016;16(1):1193.

For peer review only

For peer review only - http://bmjopen.bmj.com/site/about/guidelines.xhtml

27. Holmgren K, Hensing G, Bültmann U, et al. Does early identification of work-related stress, combined with feedback at GP-consultation, prevent sick leave in the following 12 months? a randomized controlled trial in primary health care. *BMC Public Health* 2019;19(1):1110.

28. Social insurance in Sweden. Stockholm: Government Offices of Sweden, Ministry of Health and Social Affairs, 2016. https://www.government.se/495457/globalassets/government/dokument/socialdeparteme ntet/socialinsuranceinsweden_august-2016.pdf

29. Kaiser CP. What do we know about employee absence behavior? An interdisciplinary interpretation. *J Socio-Econ* 1998;27(1):79-96.

30. Johansson G, Lundberg I. Adjustment latitude and attendance requirements as determinants of sick leave or attendance. Empirical tests of the illness flexibility model. *Soc Sci Med* 2004;58(10):1857-68.

31. Kaldo V, Lundin A, Hallgren M, et al. Effects of internet-based cognitive behavioural therapy and physical exercise on sick leave and employment in primary care patients with depression: Two subgroup analyses. *Occup Environ Med* 2018;75(1):52-58.

32. Wikberg C, Westman J, Petersson EL, et al. Use of a self-rating scale to monitor depression severity in recurrent GP consultations in primary care - does it really make a difference? A randomised controlled study.(Report). *BMC Fam Pract* 2017;18(1).

33. Rantonen J, Karppinen J, Vehtari A, et al. Effectiveness of three interventions for secondary prevention of low back pain in the occupational health setting - A randomised controlled trial with a natural course control. *BMC Public Health* 2018;18(1)

34. MacDonald S, Maxwell M, Wilson P, et al. A powerful intervention: General practitioners'; Use of sickness certification in depression. *BMC Fam Pract* 2012;13(1):82.

35. Danielsson L, Elf M, Hensing G. Strategies to keep working among workers with common mental disorders–a grounded theory study. *Disabil Rehabil* 2019;41(7):786-95.

For peer review only - http://bmjopen.bmj.com/site/about/guidelines.xhtml

For peer review only

36. Rankin L, Fowler CJ, Stålnacke BM, et al. What influences chronic pain management? A best–worst scaling experiment with final year medical students and general practitioners. *Br J Pain* 2019;13(4):214-25.

37. de Kock CA, Lucassen PLBJ, Bor H, et al. Training GPs to improve their management of work-related problems: Results of a cluster randomized controlled trial. *Eur J Gen Pract* 2018;24(1):258-65.

38. Wikberg C, Pettersson A, Westman J, et al. Patients' perspectives on the use of the Montgomery-Asberg depression rating scale self-assessment version in primary care. *Scand J Prim Health Care* 2016;34(4):434-42.

39. Talib TL, DeChant P, Kean J, et al. A qualitative study of patients' perceptions of the utility of patient-reported outcome measures of symptoms in primary care clinics. *Quality of Life Research* 2018;27(12):3157-66.

40. Lampe L, Shadbolt N, Starcevic V, et al. Diagnostic processes in mental health: GPs and psychiatrists reading from the same book but on a different page. *Australas Psychiatry* 2012;20(5):374-78.

41. Hagger MS, Chatzisarantis NLD. Integrating the theory of planned behaviour and self-determination theory in health behaviour: A meta-analysis. *Br J Health Psychol* 2009;14(2):275-302.

42. Thorsen O, Hartveit M, Johannessen JO, et al. Typologies in GPs' referral practice. *BMC Fam Pract* 2016;17(1).

43. Pettersson A, Björkelund C, Petersson E. To score or not to score: A qualitative study on GPs views on the use of instruments for depression. *Fam Pract* 2014;31(2):215-21.

44. Patsopoulos NA. A pragmatic view on pragmatic trials. *Dialogues Clin Neurosci* 2011;13(2):217.

45. Hensing G, Alexanderson K, Bjurulf P, et al. How to measure sick leave? Literature review and suggestion of five basic measures. *Scand J Public Health* 1998;26(2):133-44.

For peer review only - http://bmjopen.bmj.com/site/about/guidelines.xhtml

46. Voss M, Stark S, Alfredsson L, et al. Comparisons of self-reported and register data on sick leave among public employees in Sweden. *Occup Environ Med* 2008;65(1):61-67.

47. Thorsen SV, Flyvholm MA, Bültmann U. Self-reported or register-based? A comparison of sick leave data among 8110 public and private employees in Denmark. *Scand J Work Environ Health* 2018;44(6):631-38.

48. Liao JM, Stack CB. Annals understanding clinical research: Implications of missing data due to dropout. *Ann Intern Med* 2017;166(8):596-98.

For peer review only

For peer review only - http://bmjopen.bmj.com/site/about/guidelines.xhtml

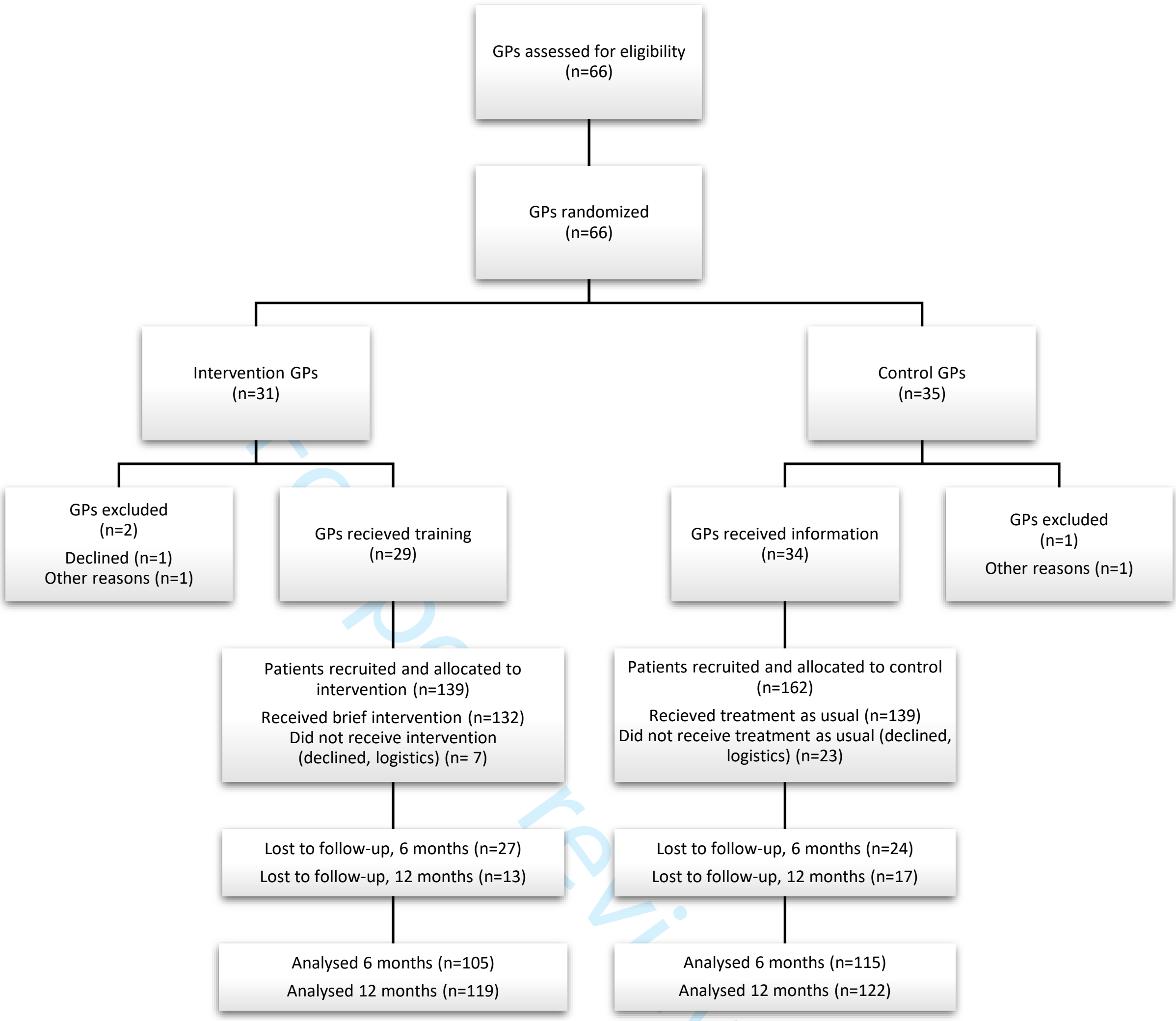

For peer review only

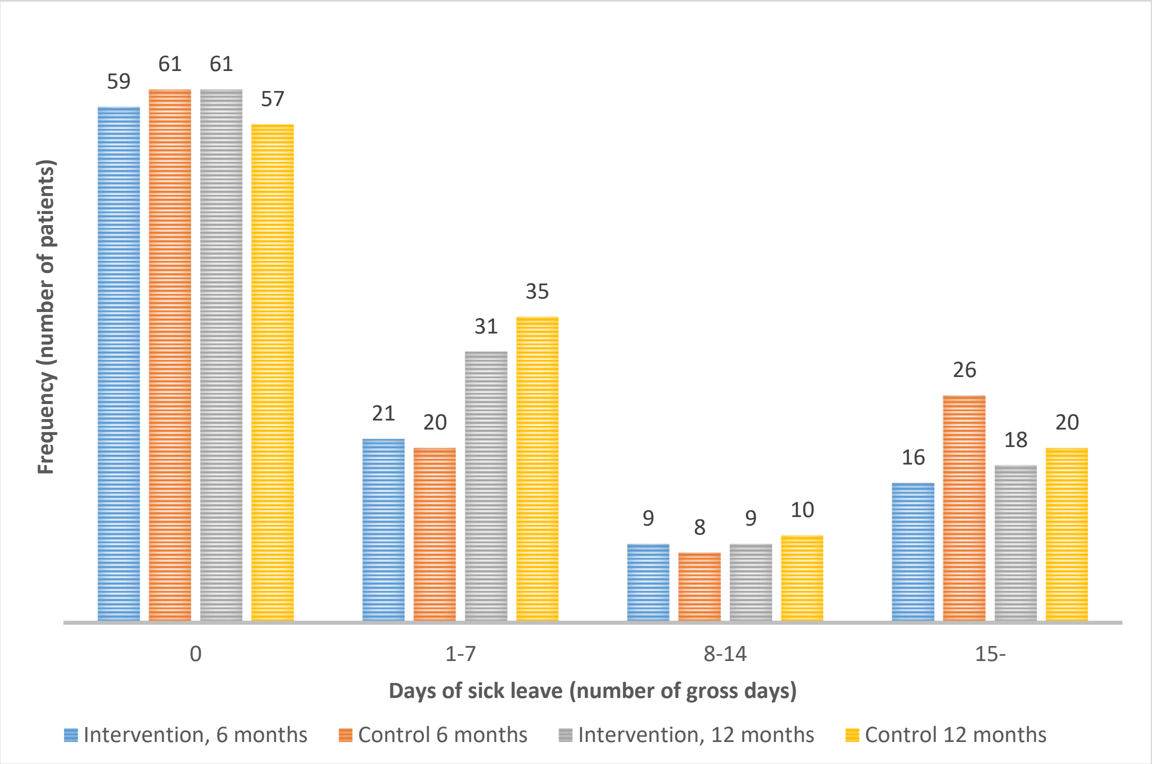
59
61
61
57
21
20
31
35
9
8
9
10
16
26
18
20
Frequency (number of patients)
0
1-7
8-14
15-
Days of sick leave (number of gross days)
Intervention, 6 months
Control 6 months
Intervention, 12 months
Control 12 months

review only

Holmgren *et al. BMC Public Health* (2016) 16:1193
DOI 10.1186/s12889-016-3852-9

BMC Public Health

STUDY PROTOCOL
Open Access

Early identification in primary health care of people at risk for sick leave due to work-related stress – study protocol of a randomized controlled trial (RCT)

Kristina Holmgren[1,2*], Christine Sandheimer[2], Ann-Charlotte Mårdby[3,4], Maria E. H. Larsson[1,5], Ute Bültmann[6], Dominique Hange[7] and Gunnel Hensing[2]

Abstract

Background: Early identification of persons at risk of sickness absence due to work-related stress is a crucial problem for society in general, and primary health care in particular. Tho date, no established method to do this exists. This project's aim is to evaluate whether systematic early identification of work-related stress can prevent sickness absence. This paper presents the study design, procedure and outcome measurements, as well as allocation and baseline characteristics of the study population.

Method/design: The study is a two-armed randomized controlled trial with follow-up at 3, 6 and 12 months. Non-sick-listed employed women and men, aged 18 to 64 years, who had mental and physical health complaints and sought care at primary health care centers (PHCC) were eligible to participate. At baseline work-related stress was measured by the Work Stress Questionnaire (WSQ), combined with feedback at consultation, at PHCC. The preventive intervention included early identification of work-related stress by the WSQ, GP training in the use of WSQ, GP feedback at consultation and finding suitable preventive measures. A process evaluation was used to explore how to facilitate future implementation and structural use of the WSQ at the PHCC. The primary outcome to compare the preventive sick leave intervention by the general practitioner (GP) versus treatment as usual is sick leave data obtained from the Swedish Social Insurance Agency register.

Discussion: Early screening for sick leave due to work-related stress makes it possible not only to identify those at risk for sick leave, but also to put focus on the patient's specific work-related stress problems, which can be helpful in finding suitable preventive measures. This study investigates if use of the WSQ by GPs at PHCCs, combined with feedback at consultation, prevents future sickness absence.

Trial registration: ClinicalTrials.gov. Identifier: NCT02480855. Registered 20 May 2015

Keywords: Psychosocial work factors, Work Stress Questionnaire (WSQ), Intervention, Organizational climate, Work commitment

* Correspondence: kristina.holmgren@neuro.gu.se
[1]Department of Health and Rehabilitation, Institute of Neuroscience and Physiology, The Sahlgrenska Academy at the University of Gothenburg, Gothenburg, Sweden
[2]Section for Epidemiology and Social Medicine, Institute of Medicine, the Sahlgrenska Academy at the University of Gothenburg, Gothenburg, Sweden
Full list of author information is available at the end of the article

BioMed Central
© The Author(s). 2016 **Open Access** This article is distributed under the terms of the Creative Commons Attribution 4.0 International License (http://creativecommons.org/licenses/by/4.0/), which permits unrestricted use, distribution, and reproduction in any medium, provided you give appropriate credit to the original author(s) and the source, provide a link to the Creative Commons license, and indicate if changes were made. The Creative Commons Public Domain Dedication waiver (http://creativecommons.org/publicdomain/zero/1.0/) applies to the data made available in this article, unless otherwise stated.

For peer review only - http://bmjopen.bmj.com/site/about/guidelines.xhtml
For peer review only

Background

Work-related stress is common in many European countries, with Sweden representing the highest level of reported work stress in Europe [1, 2]. A number of organizational and psychosocial work-related factors are found to be associated with stress, which in turn might result in adverse health effects and illness, and a higher risk of sick leave. Work-related factors, such as poor organizational climate, in terms of intolerance at work [3, 4], conflicts [5, 6], and injustice at work [7] are associated with stress, poor health and subsequent sick leave. Being engaged in work or committed to work is basically considered to have a positive influence on both the individuals' well-being and that of the organization [8, 9]. It has been demonstrated, though, that being too engaged, or over-committed, is a risk factor for sickness presenteeism [10], work-related stress [11] and poor health [12]. These organizational and psychosocial working life stressors and strains affect people negatively and result in various mental and physical health complaints, even prior to sick-listing [13–15]. People with these complaints often consult their primary health care physician [16–18] long before they even contemplate taking sick leave [11, 19]. It may well be that neither the patient, nor the general practitioner (GP) is aware that their symptoms could be caused by organizational and psychosocial factors at work. Because many patients might be at risk of disability and long-term sick leave, it is of immense value to identify these persons early and to take preventive actions [20].

Providing sickness certificates is a common task for GPs in Sweden [21, 22]. One third of Swedish GPs reported having 1–5 consultations each per week concerning sick leave [21]. This indicates that they often deal with assessing level of patients' work incapacity in their everyday practice [21, 23]. GPs often found the decision about issuing a sickness certificate difficult, especially if the patients describe symptoms without clinical findings [21, 24]. Likewise, GPs stated that they had poor knowledge of the workplace environment and the labor market [23, 25], and they reported that they barely talked to patients about their work situation [26, 27]. Today, GPs have no established practice for early identification of patients at risk for sick leave caused by adverse psychosocial factors.

The Work Stress Questionnaire (WSQ) has been designed specifically for early identification of people at risk for sick leave due to work-related stress, and was developed in the context of primary health care [11, 19, 28]. The WSQ is based on the idea that personal characteristics and environmental factors are interdependent, and that changes in either of these influences the possibilities for a sustainable work performance [29–32]. Experiences from sick-listed people [11] contributed to the questionnaire development, and showed that a poor organizational climate, as exemplified by indistinct leadership and conflicts at work, in combination with high work commitment, such as excessive individual demands and responsibility, was crucial for future sick-listing [11]. A prospective Swedish primary health care study [19] found that high stress due to poor organizational climate at baseline, measured with the WSQ, more than doubled the risk for sick leave at follow-up. Combined with high stress due to high work commitment the risk for sick leave increased fourfold.

Early screening makes it possible not only to identify patients at risk for sick leave but also to identify the patient's specific problems, which makes for the use of preventive measures and efficient treatment [33]. During the patient–GP consultation, tailored preventive measures for work-related stress can be suggested that might lower the risk of future sick leave. Since the WSQ takes both work-related factors and personal characteristics into account, it is possible to identify work-related stress from both an environmental and a personal perspective. Thus, the WSQ gives the GP the opportunity to direct preventive measures towards either the person or the workplace, or both. Therefore, it is important in GP practice to identify the patient's specific problems at work early, to communicate them to the patient, and to recommend suitable preventive measures.

Aims and hypothesis

The overall aim of this randomized controlled trial (RCT) is to evaluate whether systematic use of the WSQ, combined with feedback at consultation, can serve as a method for health care professionals in primary health care centers (PHCCs) to prevent or reduce sick leave due to work-related stress during a 12-month follow-up period. The preventive intervention will be compared versus treatment as usual (TAU). The aim is also to evaluate whether there are differences between the intervention group and the control group in healthcare measures and the prescribed medications at follow-up. In a process evaluation, the systematic use of the WSQ combined with feedback at consultation is examined.

The hypothesis of this RCT is that patients who answer the WSQ, when combined with feedback at GP consultation, will have fewer sick leave days during the year after intervention compared with those who receive TAU.

This paper presents the study design, the procedure, the outcome measurements, the allocation and the baseline characteristics of the study population. The project is still ongoing, with follow-up data to be collected and analyzes to be done. The RCT was designed in accordance with CONSORT recommendations [34].

Method and design

Study context

In Sweden, the social insurance scheme provides benefits to people who cannot work because of disease or injury.

For peer review only - http://bmjopen.bmj.com/site/about/guidelines.xhtml

Those gainfully employed are covered for the first 14 days (except for one qualification day) by their employer, and after that period benefits are granted from the Social Insurance Agency. From day 8, a medical certificate is required. Providing sickness certificates is a common task for GPs in Sweden [21, 22]. This study is conducted in PHCCs in the Västra Götaland region with a population of 1.6 million inhabitants, around 17% of the Swedish population. The region has approximately 200 public and private PHCCs with approximately 800 employed GPs.

This RCT study is part of the TIDAS project within the New Ways research program at the Section for Epidemiology and Social Medicine, Institute of Medicine, Sahlgrenska Academy, University of Gothenburg.

Study design and recruitment

This study was designed as a two-armed RCT for early identification of people at risk for sick leave due to work-related stress consulting PHCCs. The recruitment of PHCCs took place from May 2015 to November 2015. Out of the Västra Götaland region's 200 PHCCs, 51 public and private PHCCs located in rural and urban areas in and around Gothenburg were identified and consecutively invited to participate. In all, seven PHCCs (four public and three private) participated. The PHCCs were economically compensated for each participant recruited.

Randomization

GPs and residents who worked in the clinic at participating PHCCs at least 50% of the time were randomized to either the intervention or the control group. The names of all GPs at the participating PHCC were written on slips of paper that were folded and then mixed in a non-transparent bowl. Colleagues that were not involved in the RCT drew the names one at a time, and the names were alternately included in the intervention or the control group.

Procedure

Prior to the intervention period, the research team visited the participating PHCC and presented the study procedure. The control GPs were instructed to carry on as usual with their consultations. The intervention GPs received a brief training for the intervention, which included knowledge on the relationship between psychosocial factors at work, stress, health and sickness absence. GPs also received instructions on how to use, operationalize and interpret the WSQ, and on how to give feedback to the participants and refer patients at risk. Both oral and written information on the services of the primary health care specialists and occupational healthcare was presented to the GPs.

Masking (blinding)

Neither participants nor the GP were blinded to allocation in the RCT because of the nature of the intervention. All participants were given information on the study and signed consent forms before the patient–GP consultations. However, the control GPs were not informed when a patient for consultation was a study participant, and the controls filled in the questionnaires after consultation.

Eligibility to participate

Inclusion criteria

Non-sick-listed employed women and men aged 18 to 64 years who saw a GP at the PHCCs in the Västra Götaland region for mental and/or physical health complaints, including depression, anxiety, musculoskeletal disorders, gastrointestinal, cardiovascular symptoms and other stress-related symptoms were invited [16–18].

Exclusion criteria

Patients seeking care for diabetes, urinary tract infections, infections, chronic obstructive lung disease, fractures, lump and spots, allergy and psychiatric diagnoses such as schizophrenia, other psychoses or bipolar diagnoses, as well as medical check-ups were excluded. Pregnant women were also excluded because they might be at risk for pregnancy-related sick leave during the follow-up period. Patients currently on sick leave and those who had been off work for a total of 7 days or more during the last month because of sickness, with or without medical record, were excluded, as well as those with a full or part-time disability pension.

Sample size

A power calculation was performed to determine the number of participants needed to detect at least a 15% [35] difference between the intervention group and the control group concerning the primary outcome, i.e. the number of registered sick leave days (i.e. >14 days or more) during 12 months after inclusion. With a two-sided test, statistical significance of $p < 0.05$ and 80% power, at least 135 participants were needed in each group.

Data collection

Data collection took place over a period of 4–8 weeks per center (except for one center, where the data collection took 12 weeks) from May 2015 until January 2016. During data collection, a research assistant was stationed at the PHCC. The research assistant identified and recruited the eligible participants and gave oral and written information on the study. All participants were also asked to provide informed consent for the study, including linking records to registers during follow-up (Fig. 1).

For peer review only

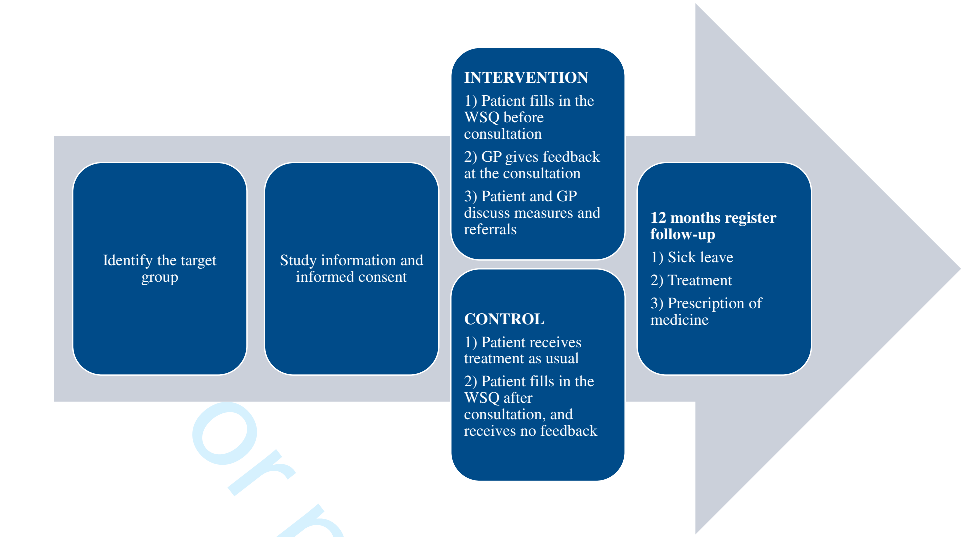

Fig. 1 The procedure of datacollection and follow-ups, TIDAS in New Ways

Intervention group

The intervention consisted of the following components: GPs' brief training in the use of the WSQ, participants' completion of the WSQ, GP feedback at consultation and finding suitable preventive measures. The WSQ consists of 21 main questions grouped into four categories [28]. Two of the categories pertain to perceived stress due to *indistinct organization and conflicts* and perceived stress due to *individual demands and commitment*. Each of these two categories contains seven items. Response options are given on a four-point ordinal scale: 'Not at all stressful', 'Less stressful', 'Stressful', and 'Very stressful'. The other two categories pertain to *influence at work* and *work interference with leisure time*, and contain four and three items, respectively, with response options given on a four-point ordinal scale: 'Yes, always', 'Yes, rather often', 'No, seldom' and 'No, never'. The reliability and face validity of the WSQ has been tested and found to be good [28].

Before the GP consultation, each participant filled in the WSQ and questions on background characteristics, which took around 15 min. The research assistant computed the WSQ and handed over the result to the GP before consultation. At the patient–GP consultation, the GPs were instructed to give feedback to the participant by communicating the results of the WSQ, and discussing possible measures, such as referrals to PHCC's specialists or to the participant's occupational healthcare (Fig. 1).

Directly after each patient–GP consultation, the GP filled in a questionnaire concerning their adherence to the instructions.

Control group

Control participants received TAU, i.e. an ordinary patient–GP consultation. The GP had no information on whether the patient was a study participant. After the GP consultation, the participant completed the WSQ and answered questions on background characteristics (Fig. 1).

Baseline assessments

Self-reported baseline characteristics were collected by questionnaire on gender (female, male), age (years), country of birth (Nordic, other), educational level (compulsory schooling, secondary school education, university or higher education), occupation, employer (private, public, self-employed), employment status (permanent, temporary, self-employed), and the reason for consulting the PHCC (mental and/or physical health complaints).

Follow-up outcome measurements

All registered data will be collected one year after last inclusion, i.e. January 2017.

Primary outcome

The number of registered sick leave days (i.e. 14 days or more) and number of absence periods during the 12 months after inclusion covered by sickness benefit will be obtained from the Swedish social insurance agency's Micro Database for Analyzing Social insurance (MiDAS) as well as data on full- and part-time sick leave and sickness and activity compensation.

Secondary outcome measurements

Short term sick leave (<14 days) and present work status are collected at 3, 6, and 12 months by telephone or email follow-up.

Healthcare measures will be obtained from the Vega database, which covers data on hospital and primary

For peer review only - http://bmjopen.bmj.com/site/about/guidelines.xhtml

health care patients in the Västra Götaland region of Sweden. Data concerning diagnoses, number of visits, referrals, and content of consultations and measures during the 12 months following inclusion.

Data on prescribed medications will be obtained from the Swedish Prescribed Drug Register, a national population-based register established in 2005, which contains information on all purchases of prescribed medications in pharmacies [36]. Data concerning the name and amount of purchased medication, date dispensed, and dosage instructions during the 12 months following inclusion.

Statistical analysis

The analyses will follow the intention-to-treat principle [37]. Per protocol analyses will be conducted to examine if deviations from the protocol have caused bias. Both descriptive and analytic statistics will be used to compare the intervention group and the control group. Analysis will be adjusted for gender and other possible confounders. Sub-group analyses will be done with regard to gender, and if possible given the number of participants, age and diagnostic groups. Non-parametric statistics will be used when ordinal data are analyzed. Otherwise, parametric statistics will be used [37].

Time plan of the RCT

The enrollment of PHCCs took place from May 2015 to November 2015. The intervention took place between May 2015 and January 2016. Follow-up of sickness absence, healthcare measures and prescribed medications in the registers will be completed one year after last inclusion, i.e. January 2017. Short-term sick leave is followed-up by telephone or e-mail at 3, 6, and 12 months until January 2017.

Process evaluation, design and procedure

Both qualitative and quantitative methods were used in the process evaluation. The target group of the process evaluation consisted of the intervention GPs. The GPs' considerations on management before, during and after intervention were assessed by questionnaires. Prior to the brief training, the GPs answered questions on readiness to use the WSQ in patient–GP consultation. Directly after each patient–GP consultation, the GP answered questions on adherence to the study protocol. After data collection, the GP answered questions on the feasibility of using the WSQ in patient–GP consultation in daily practice in the future.

After the baseline data collection was completed at each PHCC, all intervention GPs at that particular PHCC were invited to focus group discussions that explored the GPs' perception of the systematic use of the WSQ. Oral and written information was given and informed consent for the focus group study was provided. The group sessions were held at the PHCC and were moderated by a researcher experienced in focus group methodology. The discussions focused on the following key questions: views on the content of the intervention, how to improve the process, views on the readiness to use the WSQ combined with feedback in daily practice, and how to facilitate future implementation and permanent use of the WSQ at the PHCCs. The group sessions were audio taped, transcribed verbatim and analyzed according to the method of Krueger [38].

Allocation and baseline characteristics

In total, 66 GPs were randomized to either the intervention group or to the control group. One GP declined participation and two GPs were excluded because of not having the target group at consultation. The intervention group (systematic use of the WSQ and feedback during patient–GP-consulting) consisted of $n = 29$ GPs and the control group (TAU) of $n = 34$ GPs (Fig. 2).

During the inclusion period, 301 non-sick-listed employed women and men aged 18 to 64 years who sought care at the seven participating PHCCs in the Västra Götaland region and fulfilled the inclusion criteria were asked to participate in the study. Of these, 20 eligible patients (7%) declined to participate. A total of 10 patients (3%) were excluded because they left the PHCC before being asked to fill in the questionnaires. No statistically significant differences

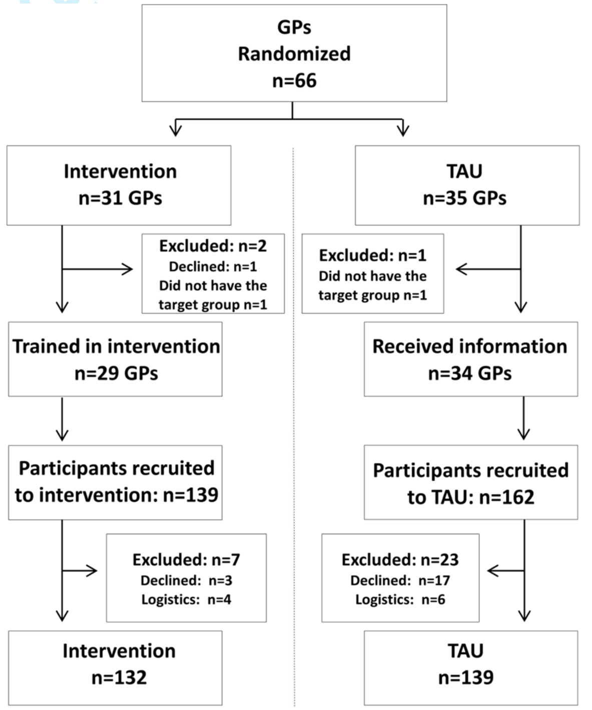

Fig. 2 Flow chart of enrolment, allocation and baseline, TIDAS in New Ways

For peer review only - http://bmjopen.bmj.com/site/about/guidelines.xhtml

in responses by participant age or gender were found. The final study population consisted of 271 participants (Fig. 2), of which 132 belonged to the intervention group and 139 to the control group.

The mean age was 46 years (standard deviation = 12) in the intervention group and 43 years (standard deviation = 11) in the control group, with a larger proportion in the age group 51–64 among intervention participants. Also, a larger proportion in the intervention group was consulting the PHCC for musculoskeletal reasons. Otherwise, there were no statistically significant differences between the groups in terms of baseline characteristics concerning sociodemographic factors and reasons for consulting the PHCC (Table 1).

Table 1 Characteristics of participants in the intervention and control groups, $n = 271$, TIDAS in New Ways

	Intervention $n = 132$	Control $n = 139$
	n[a] (%)	n[a] (%)
Gender		
Female	88 (67)	97 (70)
Age categories		
19–30 years	21 (16)	26 (19)
31–50 years	58 (44)	76 (54)
51–64 years	53 (40)	37 (27)[c]
Birthplace		
Nordic countries	122 (93)	125 (90)
Other	9 (7)	14 (10)
Educational level		
Compulsory schooling	13 (10)	15 (11)
Secondary school	61 (46)	59 (42)
University or higher	57 (44)	65 (47)
Occupational class		
Skilled/unskilled manual	49 (37)	58 (42)
Medium/low non-manual	60 (46)	56 (41)
High-level non-manual	23 (17)	24 (17)
Employer		
Private	61 (46)	68 (49)
Public	66 (50)	61 (44)
Self-employed	5 (4)	9 (7)
Reason for consultation[b]		
Mental or behavioral	75 (57)	69 (50)
Musculoskeletal	62 (47)	44 (32)[c]
Gastrointestinal	26 (20)	28 (20)
Cardiovascular	16 (12)	16 (16)
Other	29 (22)	27 (19)

[a]Dispersed numbers of participants are owing to internal missing data
[b]Multiple responses were optional
[c]Statistically significant differences (tested with the 95% CI for difference in proportion)

Discussion

There is a high level of sickness absence in Sweden, and stress-inducing factors at work play a large part in the sickness absence rate. Major efforts have been made to reduce sickness absence by restricting the sickness insurance scheme, by introducing monetary incentives to health care providers, and by specific recommended interventions, such as multimodal intervention and behavioral therapy [39]. However, none of these measures had long-term effects [39, 40]. Preventing and reducing sickness absence is challenging, and new measures are needed. Prolonged exposure to adverse psychosocial work conditions can cause stress, which in turn can lead to poor health. This scenario constitutes an obvious risk for people to be sick-listed [41–43]. People turn to their PHCCs to get help. The GPs, though, report little knowledge of work-related factors [21, 24], and rarely talk to their patients about organizational and psychosocial work-related factors [26, 27]. It is, however, essential to identify the patient at risk of being sick-listed at an early stage. This enables the GPs to take appropriate measures preventing health problems and subsequent sick leave. To date, no method exists that can be used in primary health care to identify people at risk for sick-listing due to work-related stress.

Up to now, many interventions have focused on treatment and rehabilitation of individuals already on sick leave. This is very important, but preventing sick leave is better still. Once a person is sick-listed, the return-to-work process is very costly, and this shows that much is to be gained from early identification. The focus of this project very much corresponds to needs expressed by individuals as well as society as a whole. This study is expected to show if early identification of work-related stress, using the WSQ, combined with feedback at consultation, can serve as a method for health care professionals in PHCCs to prevent or reduce sickness absence over a 12-month follow-up.

Fortunately, we reached our target sample size for participating patients. Also, the fact that few sociodemographic differences were identified between the groups was an advantage. The intervention participants were somewhat older and had a higher rate of musculoskeletal complaints as reasons for consultation. The rates of patients declining and being excluded from participation were low, and no differences concerning gender and age were observed. A limitation is that we did not collect data on non-participation patients' reasons for consultation or reasons to decline participation because of ethical considerations.

For peer review only

The advantages of randomizing at the GP level were considered as twofold: the risk for variations in sociodemographic and socioeconomic factors between participating patients in intervention and controls were reduced, and engaging the whole PHCC to recruit both to intervention and control groups led to more participants attending in earlier studies [44]. The disadvantage of randomizing at the GP level was the risk for contamination, because the GPs might discuss the study procedure with each other. Because the inclusion period was short, and the intervention was brief and imbedded in ordinary daily practice, the contamination risk was considered rather low.

A strength of this project is that both qualitative and quantitative methods were used in the process evaluation. The focus group methodology involves group discussions and is distinguished from other qualitative group interviews by the explicit use of group interaction to collect data on a specific research topic. Communication between the participating focus group members is decisive for the outcome and the group process encourages the participants to clarify not only what they think, but also how and why they think in a certain way [38, 45]. An experienced group leader was chosen to moderate the sessions because the role of the group leader is essential in creating an open and friendly atmosphere that makes participants feel free to express their views [45]. In addition to using the questionnaires on GPs' readiness and feasibility in the process evaluation, they will be analyzed in relation to the outcome variables.

Acknowledgements
We would like to thank Robin Fornazar for help with the data management, and Thorbjörn Jonsson for linguistic advice.

Funding
This study was funded by the Swedish Research Council for Health, Working Life and Welfare.

Availability of data and materials
The datasets generated and analyzed during the current study are not publicly available due ethical grounds but are available from the corresponding author on reasonable request.

Authors' contributions
KH is the principal investigator and in charge of the project. KH, A-CM, ML, UB, GH were all involved in designing the RCT and the process evaluation, and applying for funding. KH, CS and DH were primarily responsible for the data collection. KH prepared the initial draft of the manuscript and the other authors contributed. All the authors have critically reviewed and approved the final version of the manuscript.

Competing interests
A-CM was employed by Sahlgrenska University Hospital when the study was conducted (1 August 2012 to 22 May 2016) and is currently employed by Novo Nordisk A/S (23 May 2016-ongoing). The authors declare that they have no competing interests.

Consent for publication
Not applicable.

Ethics approval and consent to participate
Ethical approval was obtained from the Regional Ethical Review Board at the University of Gothenburg, Sweden, with the reference number 125–15. Informed written consent was obtained from all participants and GPs, and both the oral and written information stresses that participation was voluntary and could be terminated without any further consequences. The data is managed carefully, analyses are done only at the group level, and results are presented so that individuals cannot be recognized. The project complies with the ethical principles of the World Medical Association's Declaration of Helsinki.

Author details
[1]Department of Health and Rehabilitation, Institute of Neuroscience and Physiology, The Sahlgrenska Academy at the University of Gothenburg, Gothenburg, Sweden. [2]Section for Epidemiology and Social Medicine, Institute of Medicine, the Sahlgrenska Academy at the University of Gothenburg, Gothenburg, Sweden. [3]The Sahlgrenska University Hospital, Gothenburg, Sweden. [4]Novo Nordisk A/S, Box 50587SE-202 15 Malmö, Sweden. [5]Närhälsan Research and Development, Primary Health Care, Region Västra Götaland, Sweden. [6]Department of Health Sciences, Community and Occupational Medicine University of Groningen, University Medical Center Groningen, Groningen, The Netherlands. [7]The unit of Primary Health Care, Institute of Medicine, The Sahlgrenska Academy at the University of Gothenburg, Gothenburg, Sweden.

Received: 12 October 2016 Accepted: 17 November 2016
Published online: 25 November 2016

References

1. de Smet P, Sans S, Dramaix M, Boulenguez C, de Backer G, Ferrario M, et al. Gender and regional differences in perceived job stress across Europe. Eur J Public Health. 2005;15(5):536–45.
2. Milczarek M, Schneider E, González E. European Agency for Safety and Health at Work. OSH in figures: stress at work – facts and figures. Luxembourg: European Communities; 2009.
3. Piirainen H, Rasanen K, Kivimaki M. Organizational climate, perceived work-related symptoms and sickness absence: a population-based survey. J Occup Environ Med. 2003;45(2):175–84.
4. Arnetz BB, Lucas T, Arnetz JE. Organizational climate, occupational stress, and employee mental health: mediating effects of organizational efficiency. J Occup Environ Med. 2011;53(1):34–42.
5. Holmgren K, Dahlin-Ivanoff S, Bjorkelund C, Hensing G. The prevalence of work-related stress, and its association with self-perceived health and sick-leave, in a population of employed Swedish women. BMC Public Health. 2009;9:73.
6. Voss M, Floderus B, Diderichsen F. Physical, psychosocial, and organisational factors relative to sickness absence: a study based on Sweden Post. Occup Environ Med. 2001;58(3):178–84.
7. Kivimaki M, Elovainio M, Vahtera J, Ferrie JE. Organisational justice and health of employees: prospective cohort study. Occup Environ Med. 2003; 60(1):27–33.
8. Bakker AB, Schaufeli W, Leiter M, Taris TW. Work engagement: An emerging concept in occupational health psychology. Work Stress. 2008;22(3):187–200.
9. Bakker AB, Albrecht SL, Leiter MP. Key questions regarding work engagement. Eur J Work Organ Psy. 2011;20(1):4–28.
10. Love J, Grimby-Ekman A, Eklof M, Hagberg M, Dellve L. "Pushing oneself too hard": performance-based self-esteem as a predictor of sickness presenteeism among young adult women and men–a cohort study. J Occup Environ Med. 2010;52(6):603–9.
11. Holmgren K, Dahlin IS. Women on sickness absence – views of possibilities and obstacles for returning to work. A focus group study. Disabil Rehabil. 2004;26(4):213–22.
12. Niedhammer I, Chastang JF, David S, Barouhiel L, Barrandon G. Psychosocial work environment and mental health: Job-strain and effort-reward imbalance models in a context of major organizational changes. Int J Occup Environ Health. 2006;12(2):111–9.
13. Bultmann U, Huibers MJ, van Amelsvoort LP, Kant I, Kasl SV, Swaen GM. Psychological distress, fatigue and long-term sickness absence: prospective results from the Maastricht Cohort Study. J Occup Environ Med. 2005;47(9):941–7.
14. Stansfeld SA, Shipley MJ, Head J, Fuhrer R, Kivimaki M. Work characteristics and personal social support as determinants of subjective well-being. PLoS One. 2013;8(11):e81115.

For peer review only

15. Tynes T, Johannessen HA, Sterud T. Work-related psychosocial and organizational risk factors for headache: a 3-year follow-up study of the general working population in Norway. J Occup Environ Med. 2013;55(12): 1436–42.
16. Burton C. Beyond somatisation: a review of the understanding and treatment of medically unexplained physical symptoms (MUPS). Br J Gen Pract. 2003;53(488):231–9.
17. Toft T, Fink P, Oernboel E, Christensen K, Frostholm L, Olesen F. Mental disorders in primary care: prevalence and co-morbidity among disorders. results from the functional illness in primary care (FIP) study. Psychol Med. 2005;35(8):1175–84.
18. Reid S, Wessely S, Crayford T, Hotopf M. Medically unexplained symptoms in frequent attenders of secondary health care: retrospective cohort study. BMJ. 2001;322(7289):767.
19. Holmgren K, Fjallstrom-Lundgren M, Hensing G. Early identification of work-related stress predicted sickness absence in employed women with musculoskeletal or mental disorders: a prospective, longitudinal study in a primary health care setting. Disabil Rehabil. 2013;35(5):418–26.
20. Linton SJ, Gross D, Schultz IZ, Main C, Cote P, Pransky G, et al. Prognosis and the identification of workers risking disability: research issues and directions for future research. J Occup Rehabil. 2005;15(4):459–74.
21. Lofgren A, Hagberg J, Arrelov B, Ponzer S, Alexanderson K. Frequency and nature of problems associated with sickness certification tasks: a cross-sectional questionnaire study of 5455 physicians. Scand J Prim Health Care. 2007;25(3):178–85.
22. Soderberg E, Alexanderson K. Sickness certificates as a basis for decisions regarding entitlement to sickness insurance benefits. Scand J Public Health. 2005;33(4):314–20.
23. Pransky G, Katz JN, Benjamin K, Himmelstein J. Improving the physician role in evaluating work ability and managing disability: a survey of primary care practitioners. Disabil Rehabil. 2002;24(16):867–74.
24. Nilsen S, Werner EL, Maeland S, Eriksen HR, Magnussen LH. Considerations made by the general practitioner when dealing with sick-listing of patients suffering from subjective and composite health complaints. Scand J Prim Health Care. 2011;29(1):7–12.
25. Nilsing E, Soderberg E, Bertero C, Oberg B. Primary healthcare professionals' experiences of the sick leave process: a focus group study in Sweden. J Occup Rehabil. 2013;23(3):450–61.
26. Buijs PC, van Dijk FJ, Buijs PC, van Dijk FJ, Evers M, vd Klink JJ, Anema H. Managing work-related psychological complaints by general practitioners, in coordination with occupational physicians: a pilot study. Ind Health. 2007; 45(1):37–43.
27. Anema JR, Jettinghoff K, Houtman I, Schoemaker CG, Buijs PC, van den Berg R. Medical care of employees long-term sick listed due to mental health problems: a cohort study to describe and compare the care of the occupational physician and the general practitioner. J Occup Rehabil. 2006; 16(1):41–52.
28. Holmgren K, Hensing G, Dahlin-Ivanoff S. Development of a questionnaire assessing work-related stress in women - identifying individuals who risk being put on sick leave. Disabil Rehabil. 2009;31(4):284–92.
29. Law M, Cooper BA, Strong S, Stewart D, Rigby P, Letts L. The person-environment-occupational model: a transactive approach to occupational performance. Can J Occup Ther. 1996;63(1):9–23.
30. Dunn W, Brown C, McGuigan A. The ecology of human performance: a framework for considering the effect of context. Am J Occup Ther. 1994; 48(7):595–607.
31. Shain M, Kramer DM. Health promotion in the workplace: framing the concept; reviewing the evidence. Occup Environ Med. 2004;61(7):643–8. 585.
32. Ilmarinen J. Work ability–a comprehensive concept for occupational health research and prevention. Scand J Work Environ Health. 2009;35(1):1–5.
33. Boersma K, Linton SJ. Screening to identify patients at risk: profiles of psychological risk factors for early intervention. Clin J Pain. 2005;21(1):38–43.
34. Zwarenstein M, Treweek S, Gagnier JJ, Altman DG, Tunis S, Haynes B, et al. Improving the reporting of pragmatic trials: an extension of the CONSORT statement. BMJ. 2008;337:a2390.
35. van Beurden KM, Brouwers EP, Joosen MC, Terluin B, van der Klink JJ, van Weeghel J. Effectiveness of guideline-based care by occupational physicians on the return-to-work of workers with common mental disorders: design of a cluster-randomised controlled trial. BMC Public Health. 2013;13:193.
36. Wettermark B, Hammar N, Fored CM, Leimanis A, Otterblad Olausson P, Bergman U, et al. The new Swedish prescribed drug register–opportunities for pharmacoepidemiological research and experience from the first six months. Pharmacoepidemiol Drug Saf. 2007;16(7):726–35.
37. Altman D. Practical statistics for medical research. Boca Raton: CRC Press; 1999.
38. Krueger RA. Focus group. A practical guide for applied research. California: Sage Publications; 1988.
39. Aronsson G, Lundberg U. Rehabilitering och samordning. Ett regeringsuppdrag om att utvärdera, sammanställa och analysera resultat från forskningsprogrammet Rehsam (S2014/5621/SF). Stockholm: FORTE;2015.
40. Ejeby K, Savitskij R, Ost LG, Ekbom A, Brandt L, Ramnero J, et al. Symptom reduction due to psychosocial interventions is not accompanied by a reduction in sick leave: results from a randomized controlled trial in primary care. Scand J Prim Health Care. 2014;32(2):67–72.
41. Mather L, Bergstrom G, Blom V, Svedberg P. High job demands, job strain, and Iso-strain are risk factors for sick leave due to mental disorders: a prospective Swedish twin study with a 5-year follow-up. J Occup Environ Med. 2015;57(8):858–65.
42. Theorell T, Hammarstrom A, Gustafsson PE, Magnusson Hanson L, Janlert U, Westerlund H. Job strain and depressive symptoms in men and women: a prospective study of the working population in Sweden. J Epidemiol Commun H. 2014;68(1):78–82.
43. Nieuwenhuijsen K, Bruinvels D, Frings-Dresen M. Psychosocial work environment and stress-related disorders, a systematic review. Occup Med (Lond). 2010;60(4):277–86.
44. Mortenius H, Baigi A, Palm L, Fridlund B, Bjorkelund C, Hedberg B. Impact of the organisational culture on primary care staff members' intention to engage in research and development. J Health Organ Manag. 2015;29(2):234–51.
45. Dahlin Ivanoff S, Hultberg J. Understanding the multiple realities of everyday life: basic assumptions in focus-group methodology. Scand J Occup Ther. 2006;13(2):125–32.

Submit your next manuscript to BioMed Central and we will help you at every step:

- We accept pre-submission inquiries
- Our selector tool helps you to find the most relevant journal
- We provide round the clock customer support
- Convenient online submission
- Thorough peer review
- Inclusion in PubMed and all major indexing services
- Maximum visibility for your research

Submit your manuscript at www.biomedcentral.com/submit

BioMed Central

For peer review only

CONSORT 2010 checklist of information to include when reporting a randomised trial*

Section/Topic	Item No	Checklist item	Reported on page No
**Title and abstract**			
	1a	Identification as a randomised trial in the title	1
	1b	Structured summary of trial design, methods, results, and conclusions (for specific guidance see CONSORT for abstracts)	2
**Introduction**			
Background and objectives	2a	Scientific background and explanation of rationale	4
	2b	Specific objectives or hypotheses	5
**Methods**			
Trial design	3a	Description of trial design (such as parallel, factorial) including allocation ratio	5
	3b	Important changes to methods after trial commencement (such as eligibility criteria), with reasons	No changes
Participants	4a	Eligibility criteria for participants	7
	4b	Settings and locations where the data were collected	5
Interventions	5	The interventions for each group with sufficient details to allow replication, including how and when they were actually administered	6-7
Outcomes	6a	Completely defined pre-specified primary and secondary outcome measures, including how and when they were assessed	7
	6b	Any changes to trial outcomes after the trial commenced, with reasons	No changes
Sample size	7a	How sample size was determined	7
	7b	When applicable, explanation of any interim analyses and stopping guidelines	-
Randomisation:			
Sequence generation	8a	Method used to generate the random allocation sequence	8
	8b	Type of randomisation; details of any restriction (such as blocking and block size)	-
Allocation concealment mechanism	9	Mechanism used to implement the random allocation sequence (such as sequentially numbered containers), describing any steps taken to conceal the sequence until interventions wer8e assigned	8
Implementation	10	Who generated the random allocation sequence, who enrolled participants, and who assigned participants to interventions	8
Blinding	11a	If done, who was blinded after assignment to interventions (for example, participants, care providers, those	8

For peer review only - http://bmjopen.bmj.com/site/about/guidelines.xhtml

		assessing outcomes) and how	
	11b	If relevant, description of the similarity of interventions	-
Statistical methods	12a	Statistical methods used to compare groups for primary and secondary outcomes	8
	12b	Methods for additional analyses, such as subgroup analyses and adjusted analyses	9
**Results**			
Participant flow (a diagram is strongly recommended)	13a	For each group, the numbers of participants who were randomly assigned, received intended treatment, and were analysed for the primary outcome	9-10
	13b	For each group, losses and exclusions after randomisation, together with reasons	9-10
Recruitment	14a	Dates defining the periods of recruitment and follow-up	6-7
	14b	Why the trial ended or was stopped	-
Baseline data	15	A table showing baseline demographic and clinical characteristics for each group	10
Numbers analysed	16	For each group, number of participants (denominator) included in each analysis and whether the analysis was by original assigned groups	11
Outcomes and estimation	17a	For each primary and secondary outcome, results for each group, and the estimated effect size and its precision (such as 95% confidence interval)	12-13
	17b	For binary outcomes, presentation of both absolute and relative effect sizes is recommended	-
Ancillary analyses	18	Results of any other analyses performed, including subgroup analyses and adjusted analyses, distinguishing pre-specified from exploratory	13
Harms	19	All important harms or unintended effects in each group (for specific guidance see CONSORT for harms)	-
**Discussion**			
Limitations	20	Trial limitations, addressing sources of potential bias, imprecision, and, if relevant, multiplicity of analyses	16
Generalisability	21	Generalisability (external validity, applicability) of the trial findings	16
Interpretation	22	Interpretation consistent with results, balancing benefits and harms, and considering other relevant evidence	14
**Other information**			
Registration	23	Registration number and name of trial registry	2
Protocol	24	Where the full trial protocol can be accessed, if available	5
Funding	25	Sources of funding and other support (such as supply of drugs), role of funders	18

*We strongly recommend reading this statement in conjunction with the CONSORT 2010 Explanation and Elaboration for important clarifications on all the items. If relevant, we also recommend reading CONSORT extensions for cluster randomised trials, non-inferiority and equivalence trials, non-pharmacological treatments, herbal interventions, and pragmatic trials. Additional extensions are forthcoming: for those and for up to date references relevant to this checklist, see www.consort-statement.org.

For peer review only - http://bmjopen.bmj.com/site/about/guidelines.xhtml
For peer review only

Self-reported sick leave following a brief preventive intervention on work-related stress: a randomised controlled trial in primary health care

Journal:	*BMJ Open*
Manuscript ID	bmjopen-2020-041157.R1
Article Type:	Original research
Date Submitted by the Author:	17-Dec-2020
Complete List of Authors:	Hultén, Anna-Maria; University of Gothenburg Sahlgrenska Academy,<br>Bjerkeli, Pernilla; University of Skovde<br>Holmgren, Kristina; University of Gothenburg Sahlgrenska Academy
**Primary Subject Heading**:	General practice / Family practice
Secondary Subject Heading:	Medical education and training, General practice / Family practice, Mental health
Keywords:	PRIMARY CARE, OCCUPATIONAL & INDUSTRIAL MEDICINE, PUBLIC HEALTH, MEDICAL EDUCATION & TRAINING

SCHOLARONE™
Manuscripts

I, the Submitting Author has the right to grant and does grant on behalf of all authors of the Work (as defined in the below author licence), an exclusive licence and/or a non-exclusive licence for contributions from authors who are: i) UK Crown employees; ii) where BMJ has agreed a CC-BY licence shall apply, and/or iii) in accordance with the terms applicable for US Federal Government officers or employees acting as part of their official duties; on a worldwide, perpetual, irrevocable, royalty-free basis to BMJ Publishing Group Ltd ("BMJ") its licensees and where the relevant Journal is co-owned by BMJ to the co-owners of the Journal, to publish the Work in this journal and any other BMJ products and to exploit all rights, as set out in our licence.

The Submitting Author accepts and understands that any supply made under these terms is made by BMJ to the Submitting Author unless you are acting as an employee on behalf of your employer or a postgraduate student of an affiliated institution which is paying any applicable article publishing charge ("APC") for Open Access articles. Where the Submitting Author wishes to make the Work available on an Open Access basis (and intends to pay the relevant APC), the terms of reuse of such Open Access shall be governed by a Creative Commons licence – details of these licences and which Creative Commons licence will apply to this Work are set out in our licence referred to above.

Other than as permitted in any relevant BMJ Author's Self Archiving Policies, I confirm this Work has not been accepted for publication elsewhere, is not being considered for publication elsewhere and does not duplicate material already published. I confirm all authors consent to publication of this Work and authorise the granting of this licence.

For peer review only

Self-reported sick leave following a brief preventive intervention on work-related stress: a randomised controlled trial in primary health care

Anna-Maria Hultén[a]*, e-mail: anna-maria.hulten@gu.se

Pernilla Bjerkeli[a,b], e-mail: pernilla.bjerkeli@his.se

Kristina Holmgren[a], e-mail: kristina.holmgen@neuro.gu.se

[a]Unit of Occupational Therapy, Department of Health and Rehabilitation, Institute of Neuroscience and Physiology, Sahlgrenska Academy, University of Gothenburg, Box 455, 405 30 Gothenburg, Sweden

[b]Department for Public Health Research, University of Skövde, Box 408, Skövde, 541 28, Sweden

*Corresponding author

For peer review only - http://bmjopen.bmj.com/site/about/guidelines.xhtml

ABSTRACT

Objectives: To evaluate the effectiveness of a brief intervention about early identification of work-related stress combined with feedback at consultation with a general practitioner (GP) on the number of self-reported sick leave days.

Design: Randomised controlled trial. Prospective analyses of self-reported sick leave data collected between November 2015 and January 2017.

Setting: Seven primary health care centres in western Sweden.

Participants: The study included 271 employed, non-sick-listed patients aged 18–64 years seeking care for mental and/or physical health complaints. Of these, 132 patients were allocated to intervention and 139 patients to control.

Interventions: The intervention group received a brief intervention about work-related stress, including training for GPs, screening of patients' work-related stress, feedback to patients on screening results and discussion of measures at GP consultation. The control group received treatment as usual.

Outcome measures: The number of self-reported gross sick leave days and the number of self-reported net sick leave days, thereby also considering part-time sick leave.

Results: At 6 months follow-up 220/271 (81 %) participants were assessed, while at 12 months follow-up 241/271 (89 %) participants were assessed. At 6-month follow-up 59/105 (56%) in the intervention group and 61/115 (53%) in the control group reported no sick leave. At 12-month follow-up the corresponding numbers were 61/119 (51%) and 57/122 (47%) respectively. There were no statistical significant differences between the intervention group and the control group in the median number of self-reported gross sick leave days and the median number of self-reported net sick leave days.

Conclusions: The brief intervention showed no effect on the numbers of self-reported sick leave days for patients seeking care at the primary health care centres. Other actions and new types of interventions need to be explored to address patients' perceiving of ill health due to work-related

For peer review only - http://bmjopen.bmj.com/site/about/guidelines.xhtml

stress.

Trial registration number: NCT02480855, ClinicalTrials.gov.

STRENGTHS AND LIMITATIONS OF THIS STUDY

- Few previous RCTs have focused on patients' sick leave in a primary health care context.
- Using self-reported sick leave data made it possible to include the first two weeks of sick leave, which are not included in register data.
- Due to the inherent complexity in clinical trials in primary health care practice, the statistical power of the study might have been low.
- Sick leave data are not normally distributed and non-parametric tests therefore had to be used for the analysis.
- The outcome measure (sick leave days) is complex to interpret, as it is used both as an indicator for ill health and as a tool for treatment of ill health.

For peer review only

For peer review only - http://bmjopen.bmj.com/site/about/guidelines.xhtml

INTRODUCTION

Work-related stress has been in focus for decades, as it is common and affects the individual and the society in multiple ways. Depression, anxiety and musculoskeletal disorders are all possible consequences of work-related stress.[1 2] Psychosocial work conditions and work-related stress also constitute risk factors for sick leave.[1] As a consequence, almost 50% of the 3 billion EUR paid for sickness benefits in Sweden in 2018 were due to mental disorders,[3] whereof reaction to severe stress and adjustment disorders constituted half,[4] not to mention the loss of working hours and the costs for treatment and rehabilitation.

Sick leave is a common outcome measure in research. However, the relationship between spells, morbidity and health is complex, since sick leave is influenced strongly by factors other than personal health.[5-7] Hence, controversy exists about how to conceptualise sick leave in research.[6] As individual, social and economic forces jointly determine absence behaviour, aspects other than work-related stress must be considered, such as attendance motivation, absence culture and sickness benefit reform.[6-8] Even so, sick leave can be a useful measure not only of health status and functioning[9] but also of future sick leave and use of disability pension.[10 11] In addition, using self-reported sick leave data makes it possible to consider the first two weeks of absence, which are not included in the Swedish social insurance agency's register data.

Research has shown that there is a strong correlation between sick leave and work-related stress[12 13] and that early identification of persons perceiving ill health is important for preventing sick leave.[11 14] In addition, screening for interacting individual and work factors could make it possible to focus on the patient's specific problems and aid in finding suitable treatments.[15] In Sweden, primary health care is responsible for basic medical treatment, nursing, preventive work and rehabilitation that do not require the medical and technical resources of a hospital or other specialist skills.[16] Primary health care is also considered best suited for preventive work.[16] Since general practitioners (GPs) are often the first health care contact for persons having physical or mental health complaints

For peer review only - http://bmjopen.bmj.com/site/about/guidelines.xhtml

and often handle cases concerning stress and work ability,[17 18] they could be a possible starting point for preventive actions concerning ill health due to work-related stress.

Commonly, GPs working at a primary health care centre in Sweden have access to several other healthcare professionals, such as nurses, occupational therapists, physiotherapists and social workers, sometimes organised in psychosocial teams.[19] However, the proportion of GPs is lower than for most other comparable high-income countries, as are investments in other primary care resources.[20] In addition, earlier studies have shown that GPs might not have the prerequisites needed for early identification and treatment of patients perceiving ill health due to work-related stress in order to decrease sick leave.[21-23] Therefore, a brief preventive intervention was designed using the Work Stress Questionnaire (WSQ)[24 25] as a screening tool in combination with feedback at patient–GP consultations.[26]

METHOD

This two-armed non-blinded randomised controlled trial (RCT) was conducted at primary health care centres (PHCC) located in both urban and rural areas in the region Västra Götaland in Sweden. The trial has previously been described in detail in a study protocol.[26] The primary outcome measures for the RCT, i.e. the number of registered sick leave days and the number of sick leave periods during 12 months after inclusion, have previously been reported in a research article.[27] That study was based on data from a national Swedish register, whereas the present study uses self-reported data on sick leave. An important difference between the two data sources is that register data does only include information about sick leave spells that are 15 days or longer, whereas the self-reported data includes all sick leave. In addition, the evaluations of secondary outcome measures concerning healthcare treatments and prescription medication have been published in two other articles.[28 29]

Objectives

The objective of the study was to evaluate the effectiveness of the brief intervention about early identification of work-related stress combined with feedback at GP consultation on the number of

For peer review only - http://bmjopen.bmj.com/site/about/guidelines.xhtml

self-reported sick leave days. The overall hypothesis was that the intervention group would have fewer sick leave days during the year after the brief intervention compared to the control group. The assumptions behind this were that (1) taking part in an initial training session increased the GPs' knowledge on work-related stress, (2) filling in the WSQ raised the patients' awareness about their level of work-related stress through self-reflection, (3) receiving feedback on WSQ results increased the patients' motivation to address their work situation and (4) the combined effect of the training session, filling in the WSQ and receiving feedback constituted a basis for in-depth discussions on relevant measures at the GP–patient consultation.

The intervention concerned sick leave due to work-related stress. Hence, it was assumed that the effect of the intervention was higher for patients reporting high work-related stress or high exposure to stressors according to the WSQ. This group was therefore studied explicitly.

The work stress questionnaire

The work stress questionnaire is a self-assessment questionnaire developed in a primary health care context[24] and specifically designed to early identify people at risk for sick leave due to work-related stress. It has a broad scope, since it is not directed towards patients with a specific diagnosis. The questionnaire has a transactional perspective, as it takes the interdependence between personal and environmental work-related characteristics into account. The 21 questions included concern both psychosocial factors and the perceived stress thereof. The questions are classified into four dimensions: influence at work, indistinct organisation and conflicts, individual demands and commitment as well as work interference with leisure time.[24] In previous studies, the WSQ was found to identify work-related stress and to predict sick leave. [30 31] In addition, the test-retest reliability and face validity of the WSQ was found to be satisfying. [24 25]

Procedure

Seven PHCCs were included in the study, of which four were public and three were privately run. Participating GPs had to be working at least 50% of the time at the PHCC. The recruitment of patients

For peer review only - http://bmjopen.bmj.com/site/about/guidelines.xhtml

and the performance of the interventions were conducted in parallel for a period of 4–12 weeks at each PHCC from May 2015 until January 2016. Before the intervention period, the research team visited the centre to inform the staff about the study. During the intervention period, a research assistant was stationed at the PHCC to identify and recruit eligible participants, give information on the study and administer patients' informed consent. In addition, extra personnel resources were needed to perform the training session and to administer the WSQ to the patients. Self-reported characteristics concerning sex, age, occupational class, overall health assessed with SF-36[32] and reason for consultation were collected at baseline.

Intervention

As an initial step, the GPs randomised to intervention received a two-hour training session including information about work-related stress, ill health and sick leave. Instructions were also given on how to use the WSQ and how to give feedback to the participants; in addition, GPs received information on healthcare professionals available for referral. Before the GP–patient consultation, each patient filled in the WSQ and questions on background characteristics. During consultation, the intervention GPs gave feedback to the patients on the WSQ results. In addition, the GP and patient conferred about and initiated preventive measures, if needed.

Control

The GPs randomised to control were instructed to carry on as usual with their consultations and were not informed as to whether or not the patients were participating in the study. After the consultation, the control patients filled in the WSQ and gave information about background characteristics.

Outcomes

Follow-up data on self-reported sick leave were collected at 6 and 12 months after the intervention by telephone or email. At 6 months' follow-up the prior 3 months were reported, while at 12

For peer review only - http://bmjopen.bmj.com/site/about/guidelines.xhtml

months' follow-up the prior 6 months were reported. Data for the two follow-ups were treated separately in the analysis. The self-reported sick leave data was operationalized into two outcome measures: 1. Number of self-reported gross sick leave days and 2. Number of self-reported net sick leave days.

In Sweden, it is possible to have part-time sick leave while working the remaining 25, 50 or 75% of full time. In addition, the extent of the part-time sick leave can vary during a spell. For instance, it is possible to start with full time (100%) sick leave for two weeks and then to continue with 50% sick leave while working 50%. To be able to account for the effect of part-time sick leave in the analysis, the self-reported net days of sick leave was used as an outcome measure. Hence, working 50% part time and being on sick leave 50% for two days equals one net sick leave day and two gross sick leave days.

The number of gross sick leave days for each follow-up was calculated as the sum of the total number of self-reported sick leave days during the study period. The number of net sick leave days for each follow-up was calculated by multiplying the self-reported days of sick leave for each spell by the proportion of sick leave for that spell (25, 50, 75 or 100% of fulltime). The total number of net days during the study period were then summarized.

The outcome measures were based on the following request at follow-up: Define your sick leave during the latest 3 or 6 months, each period of sick leave separately (number of days with sick leave and proportion of full time with sick leave per period: 0%, 25%, 50%, 75%, 100% or varying proportion. If a participant reported varying proportions of sick leave during a spell, it was treated as 50% of full time for the entire spell.

Target group, sample size and power

Patients eligible to participate had to be employed, non-sick-listed, 18–64 years of age and seeking care for depression, anxiety, musculoskeletal disorders, gastro-intestinal, cardiovascular conditions

For peer review only - http://bmjopen.bmj.com/site/about/guidelines.xhtml

or other potentially stress related symptoms. Patients with seven days sickness absence or more during the last month were excluded as well as patients with sickness or activity benefits or ongoing pregnancy. Patients seeking care for other causes such as psychiatric conditions (e.g. schizophrenia, bipolar disorder), diabetes and urinary tract infection (UTI) were also excluded. The PHCCs were economically compensated for each participant recruited.

An a priori power analysis was performed for the primary outcome measure of the RCT, the number of registered sick leave days (15 days or more), with a two-sided test, a statistical significance of $p<0.05$ and an 80% power. To detect at least a 15% difference between the intervention group and the control group concerning the primary outcome, during 12 months after inclusion at least 135 participants were needed in each group.

Randomisation and blinding

The GPs at the participating PHCCs were randomised to either the intervention group or the control group with a 1:1 allocation. Folded slips of paper with their written names were mixed in a non-transparent bowl and subsequently drawn, one at a time, to the two groups alternately by colleagues not involved in the RCT. The patients consulting the GPs were therefore automatically allocated to either group. Due to the setup of the trial, none of the parties involved were blinded after assignment to interventions. All patients received the study information provided by the research assistant, the intervention GPs received information and training before the study started and the control GPs received information about the study but no training.

Statistical analyses

Descriptive statistics were compiled for the main baseline characteristics of the study population included in the overall sample. In addition, separate analyses were performed for the intervention group and the control group to detect any differences between the two. Pearson's chi-2 test was used to test if there were any differences between the intervention group and the control group concerning these characteristics.

For peer review only - http://bmjopen.bmj.com/site/about/guidelines.xhtml

Outcome data were missing for some patients, due to non-response at follow-up. Therefore, a comparison was made to test whether there were differences in characteristics between patients taking part at 6 and 12 months' follow-up, respectively, and the participants at baseline. Differences in gender proportion, age and health status were tested using chi-square test. As no statistically significant differences were observed, the patients taking part at the follow-up were included in the main analysis.

Descriptive statistics were compiled for the length of the gross sick leave periods, to get an overall understanding of the distribution of sick leave. For the analysis, the variable of self-reported gross sick leave days was categorised into four levels: 0, 1–7, 8–14 and 15 days and above. These categories were based on the Swedish sickness insurance scheme[33] stating that the employer pays sick pay for up to two weeks, with one qualifying day. Thereafter, sickness benefits are handled by the Swedish Social Insurance Agency. From day 8 of sickness onward, a doctor's certificate is required.

For the main analysis, a comparison between the intervention and control groups was made for the gross and net numbers of sick leave days at each follow-up (6 months and 12 months, respectively). As the distribution strongly deviated from a normal distribution, medians and quartiles were used to describe the centre and the spread of the data. The Mann-Whitney U test was used to test the difference between median values of gross and net numbers of sick leave days in the control group and the intervention group.

Additional analyses were conducted on five subsamples with patients who reported high exposure to stressors. In the subgroup analysis, the Mann-Whitney U test was used to test the difference between median number of gross sick leave days in the control group and in the intervention group. The subsamples were identified based on the results from the WSQ,[24] which were defined as follows:

1. *Low influence at work* included patients' seldom or never perceiving influence at work.

For peer review only

For peer review only - http://bmjopen.bmj.com/site/about/guidelines.xhtml

2. *High stress due to indistinct organisation and conflicts* included patients perceiving their work organisation and occurring conflicts as stressful or very stressful.
3. *High stress due to individual demands and commitment* included patients perceiving their own work demands and commitment as stressful or very stressful.
4. *High work to leisure time interference* included patients always or rather often perceiving interference between work and leisure.
5. *Effect from one subsample or more* included participants belonging to at least one of the above-described subsamples 1–4.

All answers were given on a four-point ordinal scale. A missing value in a dimension was replaced by the participant's median for that dimension, but only if there were answers to at least 50% of the questions in the dimension. The median values for each dimension were then categorised into high and low. All statistical analyses were performed in IBM SPSS Statistics 25.

Patient and public involvement statement

There was no patient or public involvement in the planning or conduct of this trial.

RESULTS

Participant flow

The 66 eligible GPs at the seven PHCCs were randomised to the intervention group or the control group, Figure 1. Since three GPs declined to participate or did not have patients fulfilling the criteria, there were 29 intervention GPs and 34 control GPs included. Following recruitment, 139 patients were allocated to the intervention group and 162 patients to the control group. Of these, 7 patients in the intervention group and 23 in the control group were excluded due to patients declining to participate or due to logistic reasons. Altogether, 271 patients received treatment (intervention n=132 and control n=139). Independent of group allocation, 51 of the 271 (19%) participating patients were lost to the 6-month follow-up and 30 of 271 (11%) to the 12-month follow-up. Of

For peer review only - http://bmjopen.bmj.com/site/about/guidelines.xhtml

these, 13 (5%) did not participate in either of the follow-ups. At 6 months' follow-up, data from 220 patients were included in the main analysis, while at 12 months' follow-up data from 241 patients were included. A flowchart for the enrolment, allocation and follow-ups is presented in Figure 1.

Insert Figure 1

Figure 1 *Flowchart of enrolment, allocation and follow up.*

Baseline data

As shown in Table 1, two thirds of the participants (185/271) were women, 50% (134/271) were between 31-50 years old and 40% (108/271) rated their health as good. The intervention group (n=132) and the control group (n=139) had similar distribution of background characteristics at baseline (n=271). However, the participants in the intervention group sought care for musculoskeletal ill health to a higher extent.

Results from the WSQ showed that 108 (40%) of the 271 participants assessed their influence at work as low, independent of group. In addition, 54 (20%) of the 271 participants reported high stress due to indistinct organisation and conflicts, while 124 (46%) reported high stress due to high individual demands and work commitment. The fourth WSQ-dimension, interference of work with leisure time, was high for 109 (40%) of the patients. Finally, 188 (69%) of the patients had stressors or stress from at least one of the four dimensions (effect from one subsample or more).

For peer review only

For peer review only - http://bmjopen.bmj.com/site/about/guidelines.xhtml

Table 1 Baseline characteristics of the 271 patients included in the randomised controlled trial and allocated to the intervention group or the control group.

Variable		Total (n=271)		Intervention (n=132)		Control (n=139)		p-value[1]
		n	(%)	n	(%)	n	(%)	
Sex	Male	86	32	44	33	42	30	0.582
	Female	185	68	88	67	97	70	
Age (years)	1830	47	17	21	16	26	19	0.060
	31–50	134	50	58	44	76	54	
	51–64	90	33	53	40	37	27	
Occupational class	Skilled/unskilled manual	107	40	49	37	58	42	0.675
	Medium/low non-manual	116	43	60	46	56	40	
	High-level non manual	47	17	23	17	24	17	
	Missing	1	0			1	1	
Overall health, self-rated[2]	Excellent/very good	77	28	34	26	43	30	0.526
	Good	108	40	53	40	55	40	
	Satisfactory/unsatisfactory	73	27	39	30	34	25	
	Missing	13	5	6	4	7	5	
Reason for consultation[3]	Mental or behavioural	144	53	75	57	69	50	0.237
	Musculoskeletal	106	39	62	47	44	32	0.010
	Gastrointestinal	54	20	26	20	28	20	0.927
	Cardiovascular	32	12	16	12	16	13	0.876
	Other	56	21	29	22	27	19	0.605
WSQ results[4]	Low influence at work	108	40	54	41	54	39	0.729
	High stress organisation/conflicts	54	20	28	21	26	19	0.626
	High stress demands/work commit.	124	46	63	48	61	44	0.561
	High work to leisure time interf.	109	40	54	41	55	40	0.860
	Effect from one subsample or more	188	69	91	69	97	70	0.809

[1]Pearson's chi-2 test to test differences between the intervention group and the control group.
[2]Short Form Health Survey, SF-36[32]
[3]More than one reason for consultation was possible
[4]Work Stress Questionnaire results from the four dimensions dichotomized into high and low levels as well as from the summary variable including effect from at least one dimension

Analysis of participants responding at follow-up

The basic characteristics of the participants in the intervention and the control groups responding at follow-up are shown in Table 2. No statistically significant differences were found between baseline and responders at 6 and 12 months' follow-up concerning sex, age or self-rated health.

Table 2 Characteristics of participants responding in the intervention group and the control group at 6 and 12 months' follow-up compared to baseline.

Variable, 6 months (n=220)		Intervention			Control		
		Baseline	Follow-up[1]	p-value[2]	Baseline	Follow-up[1]	p-value[2]
Numbers		132	105		139	115	
Sex	Male	44	33	0.756	42	35	0.970
	Female	88	72		97	80	
Age (years)	18–30	21	17	0.950	26	23	0.908
	31–50	58	44		76	64	
	51–64	53	44		37	28	

For peer review only - http://bmjopen.bmj.com/site/about/guidelines.xhtml

For peer review only

Overall health, self-rated[3]	Excellent/very good	34	26	0.807	43	36	0.988
	Good	53	39		55	45	
	Satisfactory/unsatisfactory	39	35		34	27	
	Missing	6	5		7	7	
**Variable, 12 months (n=241)**		**Intervention**			**Control**		
		**Baseline**	**Follow-up[4]**	**p-value[2]**	**Baseline**	**Follow-up[1]**	**p-value[2]**
Numbers		132	119		139	122	
Sex	Male	44	39	0.925	42	40	0.655
	Female	88	80		97	82	
Age (years)	18–30	21	20	0.951	26	24	0.869
	31–50	58	50		76	69	
	51–64	53	49		37	29	
Overall health, self-rated[3]	Excellent/very good	34	32	0.968	43	38	0.968
	Good	53	46		55	49	
	Satisfactory/unsatisfactory	39	35		34	28	
	Missing	6	6		7	7	

[1]6 months' follow-up
[2]Testing the distribution between baseline and responders at 6 months' follow-up concerning sex, age and health with Pearson's chi-2 test
[3]Short Form Health Survey, SF-36[32]
[4]12 months' follow-up

Descriptive statistics of sick leave

As shown in Figure 2, 59 (56%) of the 105 participants in the intervention group and 61 (53%) of the 115 participants in the control group reported no sick leave at all at the 6-months follow-up. At the 12-months follow-up, the corresponding numbers were 61 (51%) out of 119 and 57 (47%) out of 122, respectively. In addition, at 6-months follow-up 30 (29%) out of 105 in the intervention group and 28 (24%) out of 115 in the control group reported 1-14 days of self-reported gross sick leave (short-term sick-leave). At 12-month follow-up the corresponding numbers were 40 (34%) out of 119 in the intervention group and 45 (37%) out of 122 in the control group.

Insert Figure 2

Figure 2 *Number of patients having 0, 1-7, 8-14 and 15- gross days of sick leave at 6 months' follow-up (n=105 in the intervention group and n=115 in the control group) and at 12 months' follow-up (n=119 in the intervention group and n=122 in the control group).*

Main analysis of sick leave

The main analysis included 220 participants at 6 months' follow-up and 241 participants at 12 months' follow-up (Figure 1). As shown in Table 3, the median numbers of both gross and net sick leave days at 6 months follow-up were 0 days in the intervention group as well as in the control

For peer review only

For peer review only - http://bmjopen.bmj.com/site/about/guidelines.xhtml

300 group. At 12 months follow-up, the median numbers of both gross and net sick leave days were 0
301 days in the intervention group and 1 day in the control group. The differences were, however, not
302 statistically significant, since the p-value for gross days was 0,505 and the p-value for net days was
303 0,490.

304 **Sick leave in subsamples exposed to high levels of work related stress**

305 A comparison of the numbers of gross sick leave days for each of the five subsamples with
306 participants who reported high levels of work related stress, is shown in Table 3. The differences in
307 median number of sick leave days between the intervention group and the control group varied
308 between 0 and 2 days in the different subsamples. In all subsamples, the median number of gross
309 days with sick leave were equal or higher in the intervention group compared to the control group.
310 There were, however, no statistically significant differences between the groups (p-values are shown
311 in Table 3).

312 *Table 3 Comparison of number of sick leave days between the intervention group and the*
313 *control group at 6 and 12 months' follow-up, including analysis for five subsamples.*

Follow-up	Sick leave measure	Group	Number of sick leave days Q1[2]	Median	Q3[3]	p-value[1]
6 months, (n=220)	Gross days	Intervention	0.0	0.0	6.0	0.449
		Control	0.0	0.0	10.0	
	Net days	Intervention	0.0	0.0	5.9	0.398
		Control	0.0	0.0	9.0	
12 months, (n=241)	Gross days	Intervention	0.0	0.0	7.0	0.505
		Control	0.0	1.0	7.2	
	Net days	Intervention	0.0	0.0	6.2	0.490
		Control	0.0	1.0	6.2	
**Subsamples**	**Sick leave measure**	**Group**	**Number of sick leave days Q1[2]**	**Median**	**Q3[3]**	**p-value[1]**
Low influence	Gross days 6 months, (n= 89)	Intervention	0.0	1.0	10.0	0.810
		Control	0.0	0.5	27.0	
	Gross days 12 months (n= 94)	Intervention	0.0	2.0	7.0	0.916
		Control	0.0	2.0	6.0	
Stress due to organisation and conflicts	Gross days 6 months (n=45)	Intervention	0.0	0.0	7.5	0.931
		Control	0.0	0.0	17.5	
	Gross days 12 months (n=47)	Intervention	0.0	2.5	7.7	0.877
		Control	0.0	2.0	12.0	
Stress due to commitment	Gross days 6 months (n=103)	Intervention	0.0	1.0	14.5	0.793
		Control	0.0	0.0	10.2	
	Gross days 12 months (n=106)	Intervention	0.0	2.0	8.0	0.321
		Control	0.0	0.0	5.0	

For peer review only - http://bmjopen.bmj.com/site/about/guidelines.xhtml

Work to leisure time interference	Gross days 6 months (n=89)	Intervention	0.0	0.0	6.5	0.446
		Control	0.0	0.0	30.0	
	Gross days 12 months (n=96)	Intervention	0.0	2.0	10.0	0.296
		Control	0.0	0.0	5.0	
Effect, any dimension	Gross days 6 months (n=154)	Intervention	0.0	0.0	8.0	0.492
		Control	0.0	0.0	19.0	
	Gross days 12 months (n=14)	Intervention	0.0	2.0	8.7	0.310
		Control	0.0	1.0	5.7	

[1]Mann-Whitney U test
[2]First quartile
[2]Third quartile

DISCUSSION

Principal findings

This study investigated differences in self-reported sick leave between patients receiving a brief intervention to prevent sick leave due to work-related stress and those receiving treatment as usual. The results indicate that there was no significant difference in self-reported sick leave between the intervention group and the control group at 6 and 12 months' follow-up. This is in line with earlier findings from the same RCT using sick leave data from a national Swedish register including only sick leave periods 15 days and above.[27] Further, there were no significant differences in the subsamples, that is, among patients who reported high exposure to work related stressors.

Interpretation of findings

In this study, sick leave is used as an outcome measure, as it is considered a useful integrated measure of physical, psychological and social functioning in studies of working populations.[9] However, the relationship between ill health and sick leave is complex,[7 34] since it includes absence from work that is attributed to sickness by the employee and accepted as such by the employer[5] and other actors. To some extent, sick leave reflects employees' perceptions of their health and their behaviour in response to ill health.[9] Ill health can therefore be treated as a prerequisite of sick leave seen in relation to conditions within and outside of work.[35] Thus, previous intervention studies on sick leave have not demonstrated any effect on sick leave.[36-38] Further, short-term sick leave is considered to be more influenced by social, legal and psychological factors than health compared to long-term sick leave.[8 9] An essential component of the brief intervention was the discussion of

For peer review only

For peer review only - http://bmjopen.bmj.com/site/about/guidelines.xhtml

relevant preventive measures during consultation. In general, GPs regard sickness certification as a powerful and important tool.[39] In addition, workers use sick leave as a form of self-medication and a preventive measure when perceiving strain at work.[40] Hence, the brief intervention might have contributed to GPs and patients using short-term sick leave as an early treatment and as a preventive measure to a higher extent than otherwise. Since sick leave is used both as an indicator for ill health and as a tool for treatment of ill health, an initial reduction in sick leave might not be a positive outcome of the brief intervention. This complexity might be a reason why the number of sick leave days was not lower for the intervention group than the control group.

The layout of the brief intervention is fundamental for the results retrieved. The first and perhaps foremost aspect of the intervention was to increase the GPs' knowledge and awareness about work-related stress, but the training session received might not have been exhaustive enough to raise GPs' attention to patients with work-related problems or lead them to address such a complex health issue.[41 42] Secondly, filling in the WSQ was expected to increase the patients' awareness about their symptoms being stress-related. The use of patient-reported outcome measures has indeed been shown to improve the understanding of symptoms and facilitate communication.[43 44] However, early in the clinical reasoning process patients could be in need of rapport building and exclusion of physical diseases and consequently resist a psychiatric explanation.[45] Thirdly, receiving feedback on WSQ results was hypothesised to increase patients' motivation to address their work situation. However, the link between antecedents of motivation and enactment is complex. It is therefore necessary to take, for instance, past behaviour, intention, perceived behavioural control and outcome expectancy into account[46] to be able to understand this link. Thus, receiving feedback might not be sufficient to increase motivation to act. Fourthly, the first three components combined in the brief intervention were assumed to constitute a basis for fruitful GP–patient discussions and initiating relevant measures. In concordance, collaborations with patients and colleagues are seen as important elements in the referral process.[47] However, according to GPs, other aspects such as reluctance to cooperate with patients and sparse contact with colleagues could

For peer review only - http://bmjopen.bmj.com/site/about/guidelines.xhtml

affect the referral process[47] and the measures taken. Taken together, factors related to the study setup might have diluted the effect of the intervention, so that no difference in self-reported sick leave days was detected, even for the subsamples highly exposed to stressors.

The last step of the brief intervention, that is, discussing measures, was left for the GPs to organise as they deemed fit, rather than being specified in the study protocol. In general, GPs have a common understanding of their practice arising not only from the field of general practice but also from the mission of the Swedish primary health care system.[19] The overall way of working would therefore be similar. However, the results from a process evaluation of this RCT[48] indicate that the prerequisites for discussing measures might not have been ideal. The brief intervention was not found to assist the GPs in their work, since it could alter their already well-functioning work procedure. This confirms previous findings, where the use of instruments to obtain a quantitative score of depression was not perceived as useful by GPs.[49] The process evaluation also showed that the GPs could find it difficult to interpret and act on the results from the WSQ and could even question their responsibility for prevention of patients' ill health due to work-related stress, when resources were sparse. The intervention might therefore not have been efficient enough to add any effect on the days of sick leave at the follow-ups. Further, these aspects might have diminished the differences in measures taken between the intervention group and the control groups.

Strengths and limitations

Few RCTs in primary health care have focused on patients' sick leave.[36-38] In some respects, this study can be considered as pragmatic, since it is designed to test the impact of the brief intervention on sick leave in clinical practice. Inherent in pragmatic trials is a significant heterogeneity concerning patients, treatments and clinical settings, which leads to dilution of the effect of the intervention.[50] Consequently, pragmatic trials must be large. The initial power calculation stipulated a need for 135 individuals per group in order to detect a 15% difference between the groups. In the current study, groups with 105 and 115 participants per group at 6 months follow-up and 119 and 122 participants per group at 12 months follow up were compared. The statistical power of the study is thus

For peer review only - http://bmjopen.bmj.com/site/about/guidelines.xhtml

uncertain. It is therefore not possible to exclude the risk that there were differences between the groups that could not be detected due to lack of statistical power. However, looking more closely at the data, there are no trends that would suggest undetected differences in the main analysis. The number of days with sick leave are almost equal in the two groups regardless of outcome measure at 6 moths follow-up. At 12 months follow-up, the median number of days is slightly higher in the control group than the intervention group but the difference is small (0 versus 1) and not strongly reflected in the quartiles for any of the outcome variables. The subgroup analysis of individual who reported high exposure to work related stress was performed as an attempt to focus the analysis towards a group of participants where the effect of the intervention was expected to be more pronounced, thus requiring smaller groups in order to be statistically detected. There were, however, no statically significant differences in the subgroup analysis either. It should be noted that the non-significant differences in the subgroup analysis, all point in the same direction. In all sub-samples the median number of days with sick leave is equal or higher in the intervention group than in the control group. This non-significant trend is opposite to what was detected in the main analysis where there was a slightly higher median number of days in the control group at 12 months follow-up. The fact that none of the differences were statically significant and that the numbers point in different directions could be regarded as support for the finding that the intervention was not effective. However, the fact that all differences in the subgroup analysis pointed in the same direction could also suggest that the intervention did have effect among those who reported high exposure to work related stress but that the statistical power was too low to detect this difference. Another study design, including a larger group of individuals with known high exposure to stress would be needed to investigate this further.

The trial also included aspects of explanatory trials, that is, trials that aim to evaluate the efficacy of an intervention in a well-defined and controlled setting,[50] as extra personnel administered parts of the intervention. Otherwise, the study would not have been feasible. As a result, the generalisability and application in routine practice settings decreased.

For peer review only - http://bmjopen.bmj.com/site/about/guidelines.xhtml

The choice of outcome measures has to be taken into consideration. There are different methodological aspects and approaches to consider in using sick leave data in research.[51] Spell measures, person measures and time-based measures have to be used wisely[51] to capture any differences between the intervention group and the control group. Therefore, both the self-reported gross sick leave days and net sick leave days were used as outcome measures in this study. However, other outcome measures describing sick leave, such as not only number of days from intervention to sick leave but also health-related measures, might have been needed to capture an effect of the intervention.

The use of self-reported sick leave data was considered as a reasonable choice, as it made it possible to account for the first two weeks of sick leave. Thereby, any short periods of sick leave initiated by the workers themselves or by the GPs were included. Even so, self-reported data can be afflicted with recall bias. However, earlier studies indicate that there is good agreement between self-reported data and register information.[52 53] Even though the response rate was high, data were missing. Non-responders had to be accounted for, as this could affect the validity of trial findings.[54] Multiple imputation of missing data was not possible, since the data were not normally distributed. In addition, simple imputation, such as last value carried forward, was found to be inappropriate, as it assumes a strong correlation between a prior and a later value. Since there were no statistically significant differences in characteristics between responders at baseline and at follow-up, using not imputed data for responders at 6 and 12 months' follow-up for the main analysis was considered the best option. In addition, analysing sick leave data can be challenging, as it is not normal distributed.[50] Non-parametric tests, generally with less power, were therefore used in this study. The relatively small sample size and the statistical methods used both contributed to lowering the power. Thus, it is not possible to know whether the intervention had no effect or if it was not possible to detect an effect.

Conclusions and implications

For peer review only - http://bmjopen.bmj.com/site/about/guidelines.xhtml

Based on the results from this RCT, the brief intervention showed no effect on the number of self-reported sick leave days. The study yielded information about the provision of interventions in primary health care. When performing RCTs in primary health care settings, the design is determined by what is regarded as viable. Contextual aspects such as adapted educational efforts on different levels, the patients' needs and GPs' attitudes to the intervention have to be considered thoroughly when developing and implementing interventions on preventing sick leave due to work-related stress. In addition, the results can lead to discussions about how to use sick leave as an outcome measure. Even so, there is a significant need for further research into these issues, given the individual and societal consequences of ill health due to work-related stress and the limited resources to provide treatment in a cost-effective way.

DECLARATIONS

Acknowledgements The authors would like to acknowledge the PHCCs and especially the GPs and patients taking part in this study as well as the co-workers in the research group TIDAS for their support and feedback.

Contributors KH is the principal investigator and in charge of the RCT. KH was involved in designing the RCT and applying for funding. Statistical analysis was performed by AMH in close collaboration with PB. AMH drafted the manuscript, which was edited by KH and PB. All three authors critically reviewed and approved the final version of the manuscript.

Funding This study was funded by the Swedish Research Council for Health, Working Life and Welfare (2014-0936).

Disclaimer The Swedish Research Council for Health, Working Life and Welfare had no role in the design of the study, data collection, analysis or interpretation of data, or in writing the manuscript.

For peer review only - http://bmjopen.bmj.com/site/about/guidelines.xhtml

Competing interests The authors declare that they have no competing interests.

Patient consent for publication Not required.

Ethical approval The project received ethical approval, reference number 125–15, from the Regional Ethical Review Board in Gothenburg, Sweden.

Data availability statement For ethical reasons the datasets generated and analysed during the current study are not publicly available, but they are available from the corresponding author on reasonable request.

Word count 4152 (Introduction, Method, Results, Discussion and Conclusions and implications)

License statement Other than as permitted in any relevant BMJ Author's Self Archiving Policies, I confirm this Work has not been accepted for publication elsewhere, is not being considered for publication elsewhere and does not duplicate material already published. I confirm all authors consent to publication of this Work and authorise the granting of this licence

REFERENCES

1. Mather L, Bergström G, Blom V, et al. High job demands, job strain, and iso-strain are risk factors for sick leave due to mental disorders a prospective Swedish twin study with a 5-year follow-up. *J Occup Environ Med* 2015;57(8):858-65.

2. Da Costa BR, Vieira ER. Risk factors for work-related musculoskeletal disorders: A systematic review of recent longitudinal studies. *Am J Ind Med* 2010;53(3):285-323.

3. Socialförsäkringen i siffror 2019 [Social Insurance in Figures 2019]. Stockholm: The Swedish Social Insurance Agency, 2019.

4. Sjukskrivning för reaktioner på svår stress ökar mest [Sick leave for reactions to severe stress increases most]. Stockholm: The Swedish Social Insurance Agency, 2016.

5. Whitaker SC. The management of sick leave. *Occup Environ Med* 2001;58(6):420-24.

For peer review only - http://bmjopen.bmj.com/site/about/guidelines.xhtml

For peer review only

6. Allebeck P, Mastekaasa A. Chapter 3. Causes of sick leave: Research approaches and explanatory models. *Scand J Public Health Suppl* 2004;32(63):36-43.

7. Kangas M, Muotka J, Huhtala M, et al. Is the Ethical Culture of the Organization Associated with Sick leave? A Multilevel Analysis in a Public Sector Organization. *J Bus Ethics* 2017;140(1):131-45.

8. Aaviksoo E, Kiivet RA. Influence of the sickness benefit reform on sick leave. *Health policy* 2016;120(9):1070-78.

9. Marmot M, Feeney A, Shipley M, et al. Sick leave as a measure of health status and functioning: From the UK Whitehall II study. *J Epidemiol Community Health* 1995;49(2):124-30.

10. Kivimäki M, Forma P, Wikström J, et al. Sick leave as a risk marker of future disability pension: The 10-town study. *J Epidemiol Community Health* 2004;58(8):710-11.

11. Hultin H, Lindholm C, Malfert M, et al. Short-term sick leave and future risk of sick leave and unemployment - The impact of health status. *BMC Public Health* 2012;12(1).

12. Bakker AB, Demerouti E, de Boer E, et al. Job demand and job resources as predictors of absence duration and frequency. *J Vocat Behav* 2003;62(2):341-56.

13. Holmgren K, Hensing G, Dellve L. The association between poor organizational climate and high work commitments, and sick leave in a general population of women and men. *J Occup Environ Med* 2010;52(12):1179-85.

14. Kant I, Jansen NWH, Van Amelsvoort LGPM, et al. Structured early consultation with the occupational physician reduces sick leave among office workers at high risk for long-term sick leave: A randomized controlled trial. *J Occup Rehabil* 2008;18(1):79-86.

15. Boersma K, Linton SJ. Screening to identify patients at risk: Profiles of psychological risk factors for early intervention. *Clin J Pain* 2005;21(1):38-43.

16. God och nära vård – En primärvårdsreform [Good quality, local health care – A primary care reform]. Swedish Government Official Reports, SOU 2018:39. Stockholm: Government

For peer review only - http://bmjopen.bmj.com/site/about/guidelines.xhtml

Offices of Sweden, Ministry of Health and Social Affairs, 2018. https://data.riksdagen.se/fil/FD4442D3-C4D5-4799-869C-A00930A003F5

17. Lykkegaard J, Rosendal M, Brask K, et al. Prevalence of persons contacting general practice for psychological stress in Denmark. *Scand J Prim Health Care* 2018;36(3):272-80.

18. Lofgren A, Hagberg J, Arrelov B, et al. Frequency and nature of problems associated with sickness certification tasks: a cross-sectional questionnaire study of 5455 physicians. *Scand J Prim Health Care* 2007;25(3):178-85.

19. Primärvårdens uppdrag : en kartläggning av hur landstingens uppdrag till primärvården är formulerade. Stockholm: The National Board of Health and Welfare, 2016.

20. Anell A. The public-private pendulum-patient choice and equity in Sweden. *New Engl J Med* 2015;372(1):1-4.

21. Sturesson M, Edlund C, Fjellman-Wiklund A, et al. Work ability as obscure, complex and unique: Views of Swedish occupational therapists and physicians. *Work* 2013;45(1):117-28.

22. Nilsing E, Söderberg E, Berterö C, et al. Primary healthcare professionals' experiences of the sick leave process: A focus group study in Sweden. *J Occup Rehabil* 2013;23(3):450-61.

23. Carlsson L, Lännerström L, Wallman T, et al. General practitioners' perceptions of working with the certification of sick leaves following changes in the Swedish social security system: A qualitative focus-group study. *BMC Fam Pract* 2015;16(1)

24. Holmgren K, Hensing G, Dahlin-Ivanoff S. Development of a questionnaire assessing work-related stress in women - Identifying individuals who risk being put on sick leave. *Disabil Rehabil* 2009;31(4):284-92.

25. Frantz A, Holmgren K. The Work Stress Questionnaire (WSQ) - Reliability and face validity among male workers. BMC Public Health 2019;19(1).

26. Holmgren K, Sandheimer C, Mardby AC, et al. Early identification in primary health care of people at risk for sick leave due to work-related stress - study protocol of a randomized controlled trial (RCT). BMC Public Health 2016;16(1):1193.

For peer review only

For peer review only - http://bmjopen.bmj.com/site/about/guidelines.xhtml

27. Holmgren K, Hensing G, Bültmann U, et al. Does early identification of work-related stress, combined with feedback at GP-consultation, prevent sick leave in the following 12 months? a randomized controlled trial in primary health care. BMC Public Health 2019;19(1):1110.

28. Sandheimer C, Hedenrud T, Hensing G, et al. Effects of a work stress intervention on healthcare use and treatment compared to treatment as usual: a randomised controlled trial in Swedish primary healthcare. BMC Fam Pract. 2020 Jul 6;21(1):133.

29. Bjerkeli PJ, Skoglund I, Holmgren K. Does early identification of high work related stress affect pharmacological treatment of primary care patients? - analysis of Swedish pharmacy dispensing data in a randomised control study. BMC Fam Pract. 2020 Apr 25;21(1):70.

30. Holmgren K, Fjällström-Lundgren M, Hensing G. Early identification of work-related stress predicted sickness absence in employed women with musculoskeletal or mental disorders: a prospective, longitudinal study in a primary health care setting. Disabil Rehabil. 2013;35(5):418-426.

31. Holmgren K, Dahlin-Ivanoff S, Björkelund C, et al. The prevalence of work-related stress, and its association with self-perceived health and sick-leave, in a population of employed Swedish women. BMC Public Health. 2009;9.

32. Sullivan M, Karlsson J, Ware JE, Jr. [The Swedish SF-36 health survey, part 1] Evaluation of data quality, scaling assumptions, reliability and construct validity across general populations in Sweden. Soc Sci Med. 1995;41(10):1349.

33. Social insurance in Sweden. Stockholm: Government Offices of Sweden, Ministry of Health and Social Affairs, 2016. https://www.government.se/495457/globalassets/government/dokument/socialdepartementet/socialinsuranceinsweden_august-2016.pdf

34. Kaiser CP. What do we know about employee absence behavior? An interdisciplinary interpretation. *J Socio-Econ* 1998;27(1):79-96.

For peer review only - http://bmjopen.bmj.com/site/about/guidelines.xhtml

35. Johansson G, Lundberg I. Adjustment latitude and attendance requirements as determinants of sick leave or attendance. Empirical tests of the illness flexibility model. *Soc Sci Med* 2004;58(10):1857-68.

36. Kaldo V, Lundin A, Hallgren M, et al. Effects of internet-based cognitive behavioural therapy and physical exercise on sick leave and employment in primary care patients with depression: Two subgroup analyses. *Occup Environ Med* 2018;75(1):52-58.

37. Wikberg C, Westman J, Petersson EL, et al. Use of a self-rating scale to monitor depression severity in recurrent GP consultations in primary care - does it really make a difference? A randomised controlled study.(Report). *BMC Fam Pract* 2017;18(1).

38. Rantonen J, Karppinen J, Vehtari A, et al. Effectiveness of three interventions for secondary prevention of low back pain in the occupational health setting - A randomised controlled trial with a natural course control. *BMC Public Health* 2018;18(1)

39. MacDonald S, Maxwell M, Wilson P, et al. A powerful intervention: General practitioners'; Use of sickness certification in depression. *BMC Fam Pract* 2012;13(1):82.

40. Danielsson L, Elf M, Hensing G. Strategies to keep working among workers with common mental disorders–a grounded theory study. *Disabil Rehabil* 2019;41(7):786-95.

41. Rankin L, Fowler CJ, Stålnacke BM, et al. What influences chronic pain management? A best–worst scaling experiment with final year medical students and general practitioners. *Br J Pain* 2019;13(4):214-25.

42. de Kock CA, Lucassen PLBJ, Bor H, et al. Training GPs to improve their management of work-related problems: Results of a cluster randomized controlled trial. *Eur J Gen Pract* 2018;24(1):258-65.

43. Wikberg C, Pettersson A, Westman J, et al. Patients' perspectives on the use of the Montgomery-Asberg depression rating scale self-assessment version in primary care. *Scand J Prim Health Care* 2016;34(4):434-42.

For peer review only

44. Talib TL, DeChant P, Kean J, et al. A qualitative study of patients' perceptions of the utility of patient-reported outcome measures of symptoms in primary care clinics. *Quality of Life Research* 2018;27(12):3157-66.

45. Lampe L, Shadbolt N, Starcevic V, et al. Diagnostic processes in mental health: GPs and psychiatrists reading from the same book but on a different page. *Australas Psychiatry* 2012;20(5):374-78.

46. Hagger MS, Chatzisarantis NLD. Integrating the theory of planned behaviour and self-determination theory in health behaviour: A meta-analysis. *Br J Health Psychol* 2009;14(2):275-302.

47. Thorsen O, Hartveit M, Johannessen JO, et al. Typologies in GPs' referral practice. *BMC Fam Pract* 2016;17(1).

48. Hultén AM, Dahlin-Ivanoff S, Holmgren K. Positioning work related stress - GPs' reasoning about using the WSQ combined with feedback at consultation. BMC Fam Pract. 2020;21(1).

49. Pettersson A, Björkelund C, Petersson E. To score or not to score: A qualitative study on GPs views on the use of instruments for depression. *Fam Pract* 2014;31(2):215-21.

50. Patsopoulos NA. A pragmatic view on pragmatic trials. *Dialogues Clin Neurosci* 2011;13(2):217.

51. Hensing G, Alexanderson K, Bjurulf P, et al. How to measure sick leave? Literature review and suggestion of five basic measures. *Scand J Public Health* 1998;26(2):133-44.

52. Voss M, Stark S, Alfredsson L, et al. Comparisons of self-reported and register data on sick leave among public employees in Sweden. *Occup Environ Med* 2008;65(1):61-67.

53. Thorsen SV, Flyvholm MA, Bültmann U. Self-reported or register-based? A comparison of sick leave data among 8110 public and private employees in Denmark. *Scand J Work Environ Health* 2018;44(6):631-38.

54. Liao JM, Stack CB. Annals understanding clinical research: Implications of missing data due to dropout. *Ann Intern Med* 2017;166(8):596-98.

For peer review only - http://bmjopen.bmj.com/site/about/guidelines.xhtml

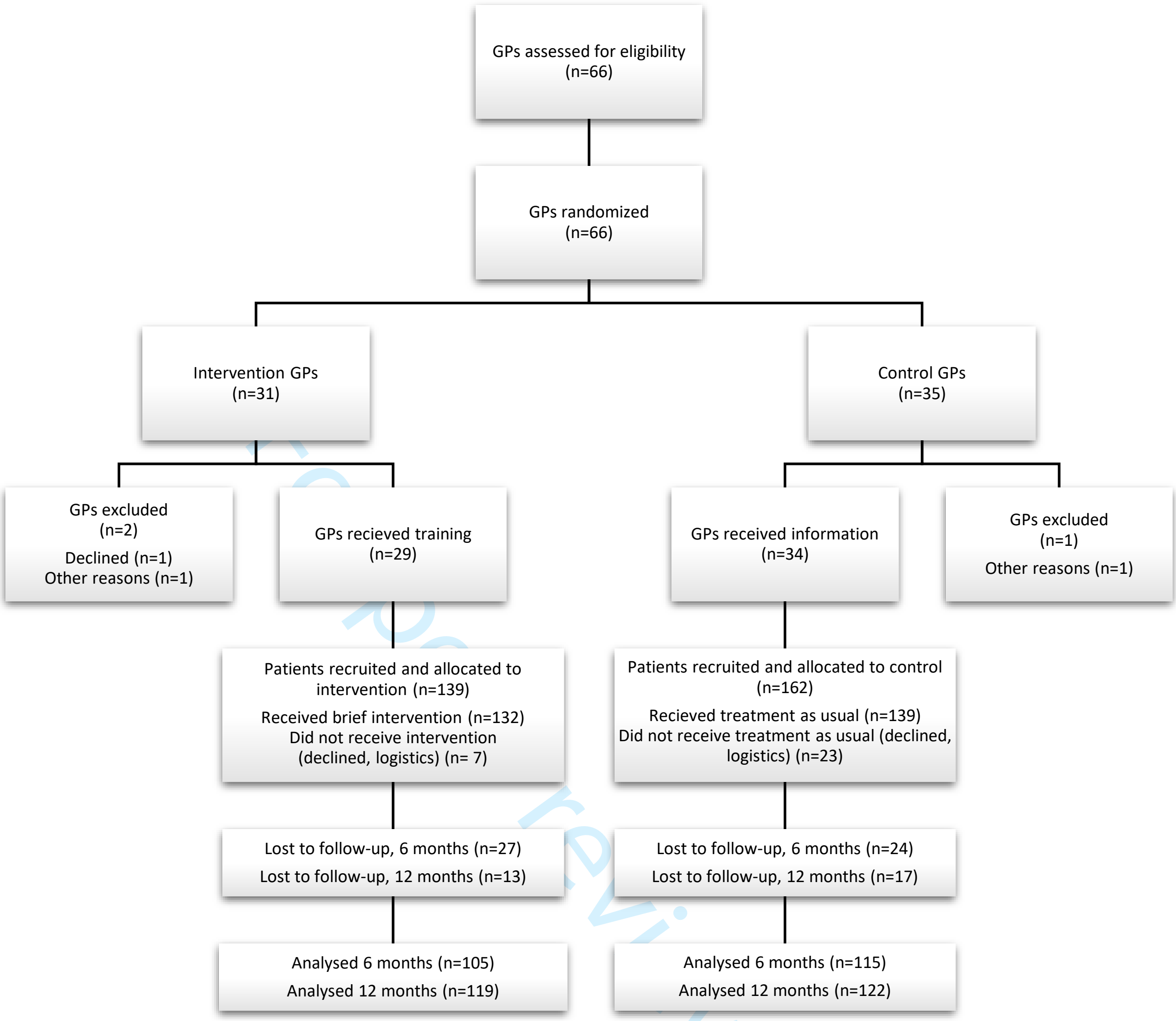
GPs assessed for eligibility (n=66)
GPs randomized (n=66)
Intervention GPs (n=31)
Control GPs (n=35)
GPs excluded (n=2)
Declined (n=1)
Other reasons (n=1)
GPs recieved training (n=29)
GPs received information (n=34)
GPs excluded (n=1)
Other reasons (n=1)
Patients recruited and allocated to intervention (n=139)
Received brief intervention (n=132)
Did not receive intervention (declined, logistics) (n= 7)
Patients recruited and allocated to control (n=162)
Recieved treatment as usual (n=139)
Did not receive treatment as usual (declined, logistics) (n=23)
Lost to follow-up, 6 months (n=27)
Lost to follow-up, 12 months (n=13)
Lost to follow-up, 6 months (n=24)
Lost to follow-up, 12 months (n=17)
Analysed 6 months (n=105)
Analysed 12 months (n=119)
Analysed 6 months (n=115)
Analysed 12 months (n=122)

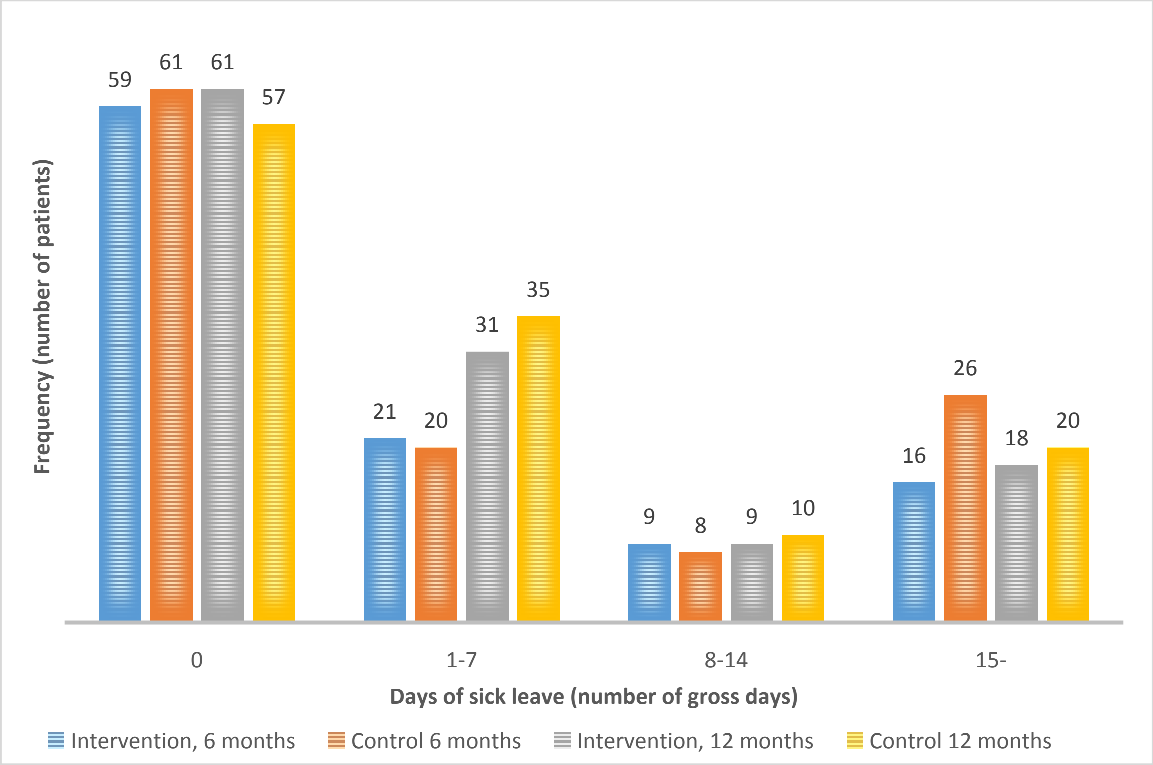

Figure 2 *Number of patients having 0, 1-7, 8-14 and 15- gross days of sick leave at 6 months' follow-up (n=105 in the intervention group and n=115 in the control group) and at 12 months' follow-up (n=119 in the intervention group and n=122 in the control group).*

review only

CONSORT 2010 checklist of information to include when reporting a randomised trial*

Section/Topic	Item No	Checklist item	Reported on page No
**Title and abstract**			
	1a	Identification as a randomised trial in the title	1
	1b	Structured summary of trial design, methods, results, and conclusions (for specific guidance see CONSORT for abstracts)	2
**Introduction**			
Background and objectives	2a	Scientific background and explanation of rationale	4
	2b	Specific objectives or hypotheses	5-6
**Methods**			
Trial design	3a	Description of trial design (such as parallel, factorial) including allocation ratio	5
	3b	Important changes to methods after trial commencement (such as eligibility criteria), with reasons	No changes
Participants	4a	Eligibility criteria for participants	8-9
	4b	Settings and locations where the data were collected	5
Interventions	5	The interventions for each group with sufficient details to allow replication, including how and when they were actually administered	7
Outcomes	6a	Completely defined pre-specified primary and secondary outcome measures, including how and when they were assessed	7-8
	6b	Any changes to trial outcomes after the trial commenced, with reasons	No changes
Sample size	7a	How sample size was determined	9
	7b	When applicable, explanation of any interim analyses and stopping guidelines	-
Randomisation:			
Sequence generation	8a	Method used to generate the random allocation sequence	9
	8b	Type of randomisation; details of any restriction (such as blocking and block size)	-
Allocation concealment mechanism	9	Mechanism used to implement the random allocation sequence (such as sequentially numbered containers), describing any steps taken to conceal the sequence until interventions wer8e assigned	9
Implementation	10	Who generated the random allocation sequence, who enrolled participants, and who assigned participants to interventions	9
Blinding	11a	If done, who was blinded after assignment to interventions (for example, participants, care providers, those	9

For peer review only

		assessing outcomes) and how	
	11b	If relevant, description of the similarity of interventions	-
Statistical methods	12a	Statistical methods used to compare groups for primary and secondary outcomes	9-10
	12b	Methods for additional analyses, such as subgroup analyses and adjusted analyses	10
**Results**			
Participant flow (a diagram is strongly recommended)	13a	For each group, the numbers of participants who were randomly assigned, received intended treatment, and were analysed for the primary outcome	11-12
	13b	For each group, losses and exclusions after randomisation, together with reasons	11-12
Recruitment	14a	Dates defining the periods of recruitment and follow-up	6-7
	14b	Why the trial ended or was stopped	-
Baseline data	15	A table showing baseline demographic and clinical characteristics for each group	13, Table 1
Numbers analysed	16	For each group, number of participants (denominator) included in each analysis and whether the analysis was by original assigned groups	11-12, Figure 1
Outcomes and estimation	17a	For each primary and secondary outcome, results for each group, and the estimated effect size and its precision (such as 95% confidence interval)	14-15
	17b	For binary outcomes, presentation of both absolute and relative effect sizes is recommended	-
Ancillary analyses	18	Results of any other analyses performed, including subgroup analyses and adjusted analyses, distinguishing pre-specified from exploratory	13-14
Harms	19	All important harms or unintended effects in each group (for specific guidance see CONSORT for harms)	-
**Discussion**			
Limitations	20	Trial limitations, addressing sources of potential bias, imprecision, and, if relevant, multiplicity of analyses	18-20
Generalisability	21	Generalisability (external validity, applicability) of the trial findings	18-20
Interpretation	22	Interpretation consistent with results, balancing benefits and harms, and considering other relevant evidence	16-18
**Other information**			
Registration	23	Registration number and name of trial registry	3
Protocol	24	Where the full trial protocol can be accessed, if available	5
Funding	25	Sources of funding and other support (such as supply of drugs), role of funders	21-22

*We strongly recommend reading this statement in conjunction with the CONSORT 2010 Explanation and Elaboration for important clarifications on all the items. If relevant, we also recommend reading CONSORT extensions for cluster randomised trials, non-inferiority and equivalence trials, non-pharmacological treatments, herbal interventions, and pragmatic trials. Additional extensions are forthcoming: for those and for up to date references relevant to this checklist, see www.consort-statement.org.

For peer review only - http://bmjopen.bmj.com/site/about/guidelines.xhtml
For peer review only
